# Systematic investigation of CO_2_ : NH_3_ ice mixtures using mid-IR and VUV spectroscopy – part 1: thermal processing†

**DOI:** 10.1039/d0ra05826b

**Published:** 2020-10-12

**Authors:** Rachel L. James, Sergio Ioppolo, Søren V. Hoffmann, Nykola C. Jones, Nigel J. Mason, Anita Dawes

**Affiliations:** School of Physical Sciences, The Open University Walton Hall Milton Keynes UK Rachel.James1@open.ac.uk +44 (0)1908 654192 +44 (0)1908 332012; School of Electronic Engineering and Computer Science, Queen Mary University of London Mile End Road London UK; ISA, Department of Physics and Astronomy, Aarhus University Ny Munkegade 120 DK-8000 Aarhus C Denmark; School of Physical Sciences, University of Kent Canterbury Kent UK

## Abstract

The adjustment of experimental parameters in interstellar ice analogues can have profound effects on molecular synthesis within an ice system. We demonstrated this by systematically investigating the stoichiometric mixing ratios of CO_2_ : NH_3_ ices as a function of thermal processing using mid-IR and VUV spectroscopy. We observed that the type of CO_2_ bonding environment was dependent on the different stoichiometric mixing ratios and that this pre-determined the NH_3_ crystallite structure after phase change. The thermal reactivity of the ices was linked to the different chemical and physical properties of the stoichiometric ratios. Our results provide new details into the chemical and physical properties of the different stoichiometric CO_2_ : NH_3_ ices enhancing our understanding of the thermally induced molecular synthesis within this ice system.

## Abstract 2

The stoichiometric mixing ratio of CO_2_ : NH_3_ ices determines both the initial chemical and physical properties of the ices and their evolution through thermal processing including CO_2_ bonding environment, NH_3_ crystallite size and amount of residue.

## Graphical abstract

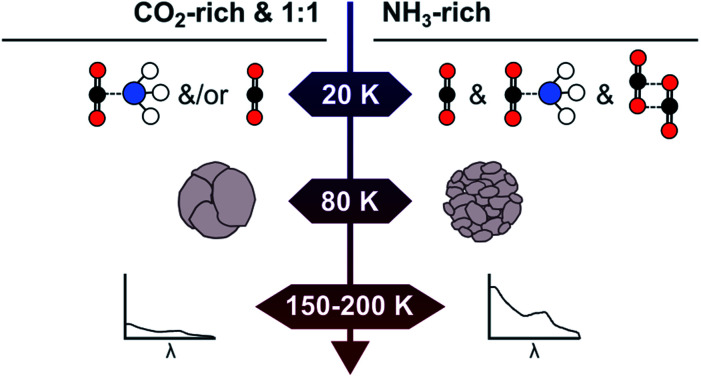

## 1 Introduction

Despite over 200 molecules being detected in the interstellar medium (ISM), the formation pathways for most of these molecules remain elusive. However, processing of interstellar ices is believed to play an important role in the molecular synthesis of the majority of these astrochemical molecules. As a result, there exists extensive experimental investigations of condensed phase molecular films under ISM conditions.

Typical to all experiments, regardless of application, a range of experimental parameters can be controlled. For astrochemical experiments investigating interstellar ice analogues, these experimental parameters include, but are not limited to, the following: deposition temperature, deposition rate, ice composition (and ratio), processing type, processing time, processing energy and substrate type. Adjusting these experimental parameters will change the chemical and physical properties of the interstellar ice analogues, the properties of which govern the molecular synthesis within the ice and requires comprehensive analysis.

To demonstrate the impact that one discrete experimental parameter can have on the chemical and physical properties of an interstellar ice system we investigated the stoichiometric mixing ratio of CO_2_ : NH_3_ ices as a function of thermal processing. We chose the CO_2_ : NH_3_ system as CO_2_ and NH_3_ are two of the most abundant molecules observed in interstellar ice and when combined contain the four most common elements to life. Furthermore, the two compounds do not share any of the elements between them, making identification of the origin of the constituents that make up the products easier, without the need for isotopic substitution. As such this is an attractive ice system to study and has been the subject of multiple previous studies which provide a benchmark to validate our systematic investigation of one discrete experimental parameter.^1–9^

Table 1 summarises the previous thermal processing studies of CO_2_ : NH_3_ interstellar ice analogues. A consensus was that a thermally induced reaction within the CO_2_ : NH_3_ ice mixture resulted in the formation of carbamic acid and/or ammonium carbamate. Of the nine experimental studies shown in Table 1 only three studies^1,6,7^ investigated the stoichiometric mixing ratio of CO_2_ : NH_3_ ice, the discrete experimental parameter under focus in this paper. And, of these three studies, CO_2_-rich mixtures were investigated by Frasco who makes no comment about the CO_2_-rich mixtures^1^ and Noble *et al.* who reported no thermal reaction in CO_2_-rich mixtures.^7^ For equal parts or NH_3_-rich mixtures Noble *et al.* observed that ammonium carbamate formed first and converted to carbamic acid at temperatures above 150 K.^7^ Rodríguez-Lazcano *et al.* reported that their CO_2_ : NH_3_ 1 : 2 ratio produced the highest yield of products compared to their 1 : 3 & 1 : 1 ratios, but offered no explanation as to why this preference existed.^6^ As such the effect of the stoichiometric mixing ratio parameter on the CO_2_ : NH_3_ ice system has potential for further investigation.

**Table 1:** Summary of the results of previous studies conducted on the thermal processing of CO_2_ : NH_3_ ice mixtures

Reference	CO_2_ : NH_3_ ratio	Deposition temperature (K)	Main products
Frasco^1^	Various (10 to 90% NH_3_)^a^	195	AC
Hisatsune^2^	1 : 3^a^	83	(NH_3_)_2_CO_2_ & AC
Bossa *et al.*^3^	1 : 1^b^	10	AC & CA
Bertin *et al.*^4^	1 : 1^a^	10	AC & CA
Lv *et al.*^5^	1 : 1^a^	16	AC & CA
Rodríguez-Lazcano *et al.*^6^	1 : 1, 1 : 2, 1 : 3^c^	15	AC & CA
Noble *et al.*^7^	Various (including 20 : 1, 13 : 1, 4 : 1, 1 : 1, 1 : 1.5)^c^	60	AC & CA
Potapov *et al.*^8^	1 : 4^c^	15	AC
Potapov *et al.*^9^	1 : 4^c^	15	AC

a Ratios derived from partial pressures of the mixture in the gas line.

b Method of determining ratio not specified.

c Ratios derived from column density.

All of the studies shown in Table 1 employed IR spectroscopy, a powerful *in situ* technique commonly used for the investigation of the structure and composition of ice samples in the laboratory. Some of these studies also incorporated a second analytical technique, *e.g.* mass spectrometry^3,7,8^ or high resolution low energy electron loss spectroscopy,^4^ providing further characterisation of the ice. In addition to using mid-IR spectroscopy to investigate the thermal processing of CO_2_ : NH_3_ ice mixtures in this paper, we also present a complementary study using vacuum-UV (VUV) spectroscopy. This is the first time that CO_2_ : NH_3_ interstellar ice analogues have been investigated using VUV spectroscopy. Both mid-IR and VUV spectroscopy are used *in situ* and provide information on the end products allowing for the monitoring of the complex chemical and physical processes involved in forming these products.

This work is part of a wider, ongoing collection of experiments aimed at understanding how discrete experimental parameters impact both the chemical and physical properties of an interstellar ice analogue which govern molecular synthesis. In particular, this paper focusses on the influence that the stoichiometric mixing ratio has on the chemical and physical properties of CO_2_ : NH_3_ mixtures when subjected to thermal processing. A corresponding paper will investigate the influence that the stoichiometric mixing ratio has on the chemical and physical properties of CO_2_ : NH_3_ mixtures when subjected to both non-thermal and thermal processing.

In this paper, we present mid-IR spectra of the thermal processing of CO_2_ : NH_3_ mixtures (3 : 1, 2 : 1, 1 : 1, 1 : 3 & 1 : 10) deposited at 20 K. We complement the mid-IR spectra with the first VUV spectroscopic study of CO_2_ : NH_3_ mixtures (4 : 1, 1 : 1 & 1 : 3) which were deposited at 20 K and thermally processed. We characterise these stoichiometric mixing ratios in detail at 20 K and demonstrate that the stoichiometric mixing ratio has a significant impact on both the chemical and physical properties of the CO_2_ : NH_3_ ice system at deposition and throughout thermal processing.

## 2 Experimental

Both the mid-IR and VUV experiments were performed using The Open University Portable Astrochemistry Chamber (PAC). The PAC set-up is described in detail in Section S1.1 of the ESI.† All samples were grown *via* physical vapour deposition onto a cooled substrate (mid-IR: ZnSe, Crystran; VUV: MgF_2_, Crystran) at a base temperature of 20 K and a base pressure of low 10^−9^ mbar. CO_2_ (99.8%, BOC) and NH_3_ (99.96%, ARGO International Ltd) were premixed in the gas line prior to deposition. The ice samples were thermally processed at a heating rate of approximately 0.1 K s^−1^ to a set temperature. The acquisition time for a mid-IR measurement was approximately 2 min. The acquisition time for a VUV measurement was dependent on the step size used, which for measurements taken at temperatures ≤ 80 K corresponded to ∼30 min and at temperatures ≥ 90 K corresponded to ∼10 min. As acquisition times were different for the mid-IR and VUV spectroscopic measurements, the samples were allowed to isothermally stabilise for 2 min before the spectroscopic measurement was taken at the set temperature.

The mid-IR experiments were performed at The Open University Molecular Astrophysics Laboratory, UK, using a FTIR spectrometer (Nicolet Nexus 670) with an external MCT detector. All mid-IR spectra were acquired in absorbance over the wavenumber range of 4000–800 cm^−1^ at a resolution of 1 cm^−1^. Background scans were averaged over 512 scans and sample scans were averaged over 128 scans. All mid-IR spectra presented in this paper were obtained at an oblique angle (45°) to the IR radiation unless otherwise stated. For the VUV experiments, the PAC was attached to the AU-UV beam line at the ASTRID2 storage ring, Aarhus University, Denmark. All VUV spectra were acquired in absorbance over the wavelength range of 120–340 nm with 0.05 to 1 nm wavelength step size depending on the width of the spectral features to be resolved. The average photon flux per point was 2 ×10^10^ photons s^−1^ and collection time per point was 2.69 s. VUV processing (*e.g.* photoionisation) is wavelength dependent and given the low average photon flux and short collection time, any VUV processing during a measurement is negligible. All VUV spectra presented in this paper were obtained at an oblique angle (45°) to the UV radiation unless otherwise stated.

All mid-IR and VUV spectra are freely available on the Open Research Data Online (ORDO) Repository.^10^

### 2.1 Film thickness

Film thickness was determined from *in situ* laser interferometry measurements using a HeNe laser beam reflected off the substrate during deposition (see Section S1.2 of the ESI† for more details). The same deposition conditions were maintained for both mid-IR and VUV experiments and the deposition rates were between 0.8–1.8 nm s^−1^. On average, the film thickness of the mid-IR samples was 402 nm. Thinner films were required to prevent saturation of absorption peaks for the VUV samples compared to mid-IR samples and the average film thickness was 204 nm. Where comparisons were made between the mixtures, the spectra were normalised to specific film thickness, 400 nm for mid-IR spectra and 200 nm for VUV spectra, and is indicated in the figure captions. See Table S1 in the ESI† for the individual sample thickness and normalisation factors.

### 2.2 Determining the CO_2_ : NH_3_ mixing ratios

It is well known that discrepancies exist between the ratio of the partial pressures in the gas line and the resultant mixing ratio of the deposited sample. For the mid-IR samples the CO_2_ : NH_3_ ratios were determined from the derived column densities of CO_2_ (*ν*_3_ absorption band) and NH_3_ (*ν*_2_ absorption band) when the substrate was positioned at normal incidence to the IR radiation. For specific details on the calculations used, integration range of the absorption peak and integrated band strengths see Section S1.3 of the ESI.† VUV spectroscopy does not have the same protocol for determining the ratios directly from the spectra as mid-IR spectroscopy. A method for determining the ratios from VUV spectra without needing to calculate photoabsorption cross sections is proposed in Section S1.4 of the ESI.† The VUV samples used the same mixing partial pressures as the mid-IR samples. While the partial pressures do not correlate with the deposited mixtures, a consistent mixing ratio was obtained from the corresponding partial pressures and this was used to check the validity of the method proposed.

## 3 Mid-IR results & discussion

### 3.1 Deposition at 20 K

**Fig. 1:**
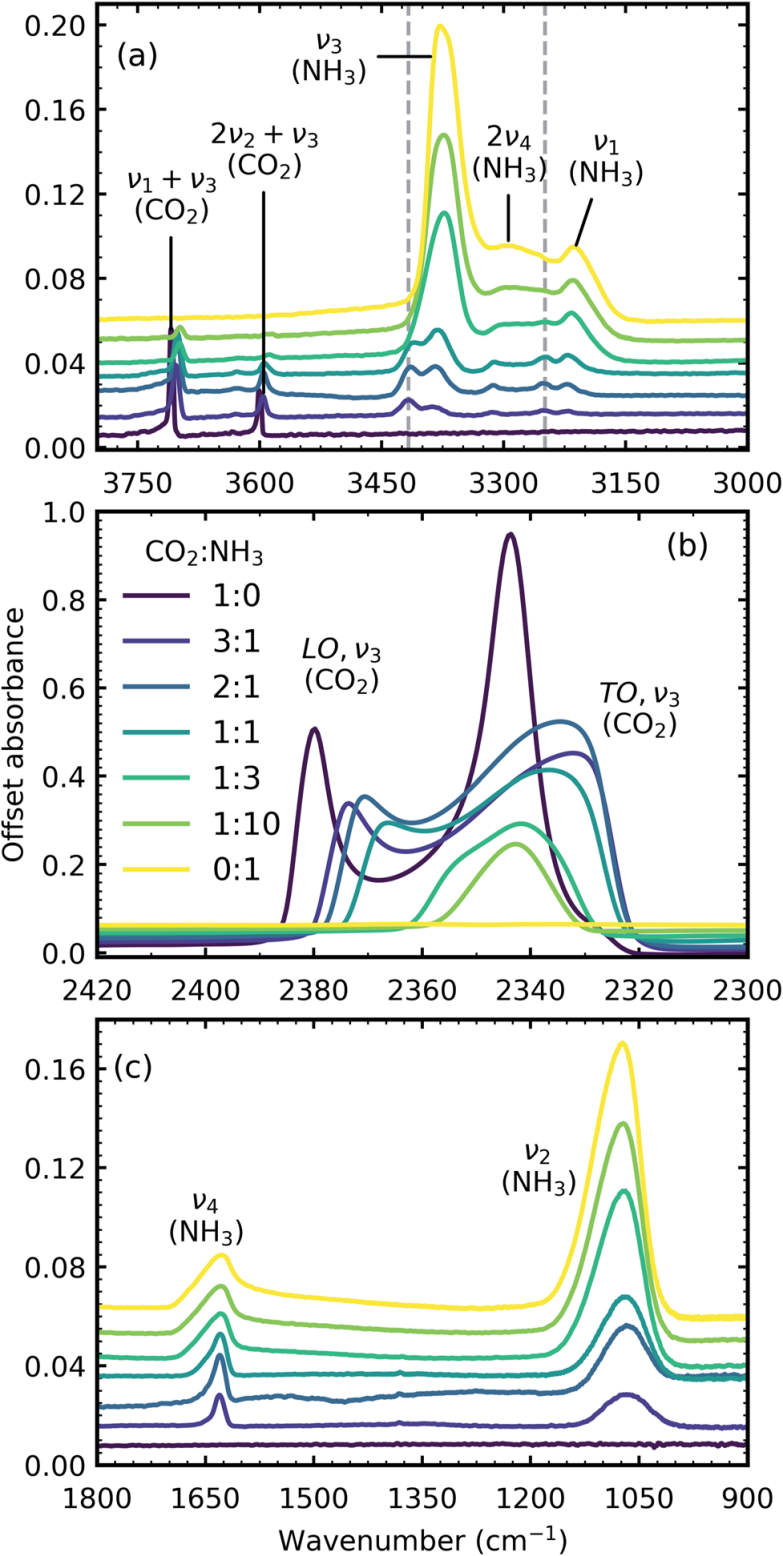
Mid-IR spectra of CO_2_ : NH_3_ mixtures (3 : 1, 2 : 1, 1 : 1, 1 : 3 & 1 : 10) at 20 K compared with pure CO_2_ (1 : 0) and pure NH_3_ (0 : 1). (a) Dashed lines represent new features which formed upon deposition. (b) LO-TO splitting of the *ν*_3_ absorption band of CO_2_ (c) NH_3_*ν*_4_ and *ν*_2_ absorption bands. Band assignments are given in Table 2. Spectra are offset on the *y*-axis for clarity. All spectra are normalised to a thickness of 400 nm.

Fig. 1 shows the mid-IR spectra of CO_2_ : NH_3_ mixtures (3 : 1, 2 : 1, 1 : 1, 1 : 3 & 1 : 10) deposited at 20 K compared with pure CO_2_ (1 : 0) and pure NH_3_ (0 : 1) also deposited at 20 K. The band assignments and positions are given in Table 2.

**Table 2:** Band assignments and positions of the vibrational modes of pure CO_2_ ice (1 : 0), pure NH_3_ ice (0 : 1) and CO_2_ : NH_3_ mixtures (3 : 1, 2 : 1, 1 : 1, 1 : 3 & 1 : 10) deposited at 20 K

Molecule	Vib. mode	Assignment	Ref.	Position (cm^−1^)						
				1 : 0	3 : 1	2 : 1	1 : 1	1 : 3	1 : 10	0 : 1
CO_2_	*ν*_1_ + *ν*_3_	Combination	11	3709	3703	3702	3701	3698	3697	
	2*ν*_2_ + *ν*_3_	Combination	11	3601	3596	3596	3595	3590	3588	
	*ν*_3_	C=O *asym*. stretch (LO)	12	2380	2374	2371	2366			
	*ν*_3_	C=O *asym*. stretch (TO)	11 and 12	2345	2332	2334	2337	2342	2343	
^13^CO_2_	*ν*_3_	*asym*. stretch	11	2283	2279	2279	2278	2277	2277	
CO_2_ : NH_3_ complex			3		3417	3415	3413			
			3		3250	3251	3248	3248		
NH_3_	*ν*_3_	N–H *asym*. stretch	13		3384	3382	3379	3373	3373	3377
	2*ν*_4_	Overtone	13		3313	3312	3311	3301	3298	3294
	*ν*_1_	N–H *sym*. stretch	13		3220	3220	3220	3215	3214	3212
	*ν*_4_ + *ν*_L_	Combination	13					1872	1872	1883
	*ν*_4_	Deformation	13		1630	1630	1629	1629	1628	1627
	*ν*_2_	Umbrella	13		1066	1067	1069	1071	1073	1073

In addition to the vibrational modes associated with NH_3_ and CO_2_ two new vibrational modes were observed in the CO_2_ : NH_3_ mixtures in Fig. 1(a) and are marked with dashed lines. The first vibrational mode at 3417 cm^−1^ was present for all ratios except the 1 : 3 and 1 : 10 ratios and the second vibrational mode at 3253 cm^−1^ was observed for all ratios except the 1 : 10 ratio. Previous studies have assigned these vibrational modes to a CO_2_ : NH_3_ molecular complex in a ‘T-shape’ whereby the NH_3_ molecule is complexed to the C atom in the CO_2_ molecule *via* the lone pair of electrons on the N atom of NH_3_.^3,14,15^

Due to the oblique angle of the ice films to the incident IR radiation, longitudinal optical (LO)-transverse optical (TO) splitting of the *ν*_3_ vibrational mode of CO_2_ was observed for pure CO_2_ ice and the 3 : 1, 2 : 1 & 1 : 1 mixtures as shown in Fig. 1b.^16,17^ LO-TO splitting arises due to long-range dipole interactions in the CO_2_ lattice and the position of the LO mode is sensitive to defects within the CO_2_ lattice.^17^ The CO_2_ in the NH_3_-rich mixtures (1 : 3 & 1 : 10) can be thought of as defects within the NH_3_ ice and hence the LO mode was not observed. The shape of the LO mode can also give insight into the mixing environment of CO_2_ mixtures. For example, Cooke *et al.* noted that the LO mode would split when inhomogeneous mixing occurred.^17^ No splitting of the LO mode was observed for 3 : 1, 2 : 1 & 1 : 1 CO_2_ : NH_3_ mixtures which suggested homogeneous mixing occurred.

#### 3.1.1 Differing absorption band shapes and positions

Broadening or narrowing of the vibrational absorption bands in the mixtures compared to the respective pure ice vibrational absorption bands were observed as shown in Fig. 1. In solids, the vibrational absorption band shapes are strongly influenced by intermolecular interactions or its local environment. Each molecule may perceive a different local bonding environment and hence, the observed vibrational absorption band is a normal distribution of oscillators around an average band intensity. Changes in the vibrational absorption band shapes of the mixtures from the pure ices reflect the differing local environments. Broadening of the absorption band suggests a wider distribution of bond lengths within the different local environments such as in a disordered matrix. Whereas, narrowing of the absorption band typically suggests an increased order such as a crystalline matrix contributing to a narrower distribution of bond lengths within the local bonding environment.

**Fig. 2:**
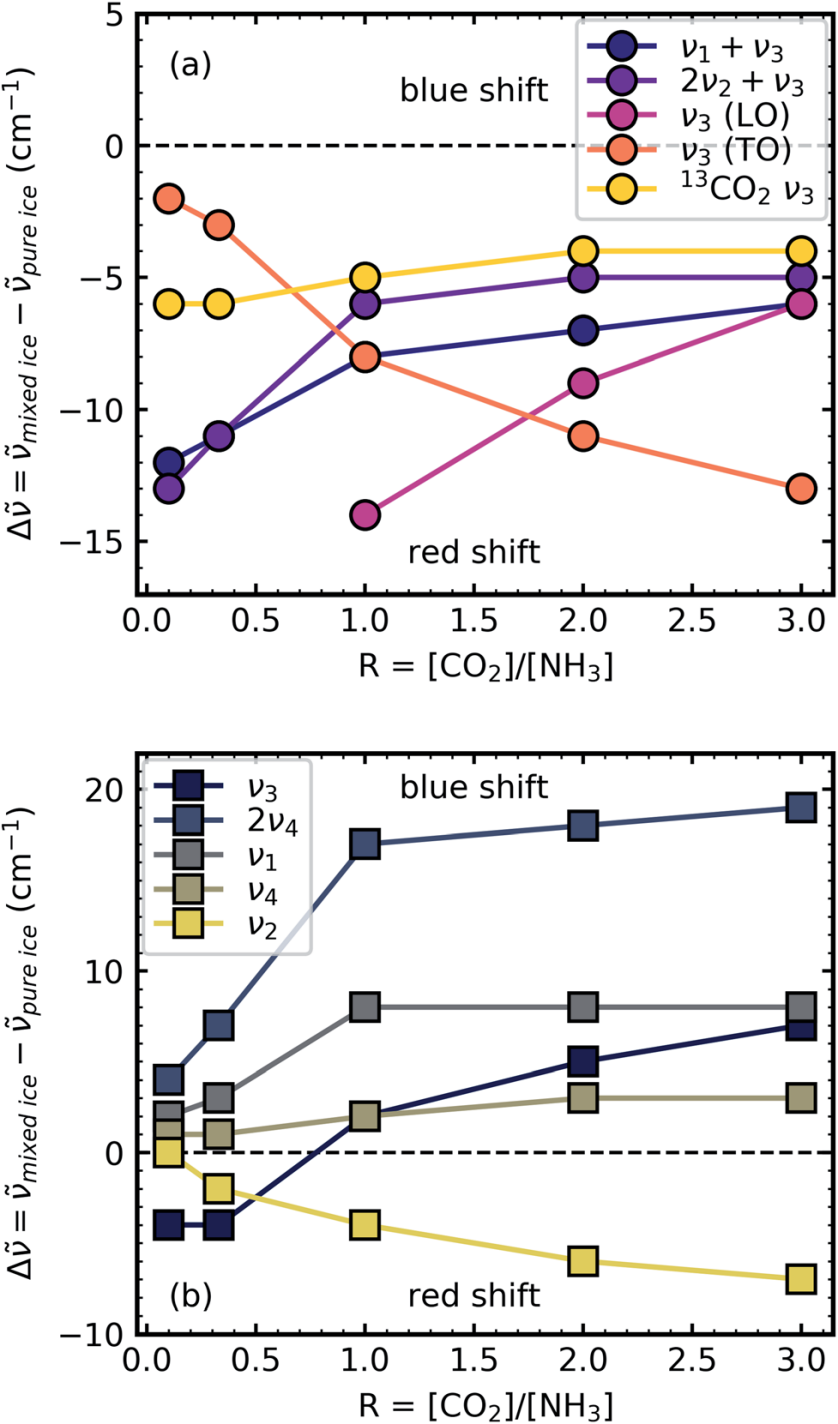
Scatter plots of the difference between the pure ice vibrational absorption band and the ice mixture vibrational absorption band (Δ*ν̃* = *ν̃*_pure ice_ − *ν̃*_mixed ice_) against the [CO_2_]/[NH_3_] ratio (R) for (a) CO_2_ vibrational absorption bands and (b) NH_3_ vibrational absorption bands. Lines between the scatter points are to guide the eye only.

Shifts in the position of the vibrational absorption bands in the mixtures compared to the respective pure ice vibrational absorption bands can also be seen in Fig. 1. A red shift is associated with an increase in bond length, and hence a weakening of the bond associated with the absorption band. The reverse is true for observed blue shifts in the spectra. Fig. 2 shows the difference between the position of the pure ice vibrational absorption band and the position of the ice mixture vibrational absorption band (Δ*ν̃* = *ν̃*_pure ice_ − *ν̃*_mixed ice_) against the [CO_2_]/[NH_3_] ratio (*R*).

All the CO_2_ vibrational absorption band positions in the mixtures (Fig. 2a) red shifted compared to the pure CO_2_ vibrational absorption bands. For all vibrational absorption bands, except for the *ν*_3_ TO mode, a progressive red shift was observed for increasing concentrations of NH_3_ (lower *R* values). This suggested a progressive weakening of the C=O bonds with increasing NH_3_ concentration. In contrast, a progressive red shift with decreasing concentrations of CO_2_ (higher *R* values) was observed for the *ν*_3_ TO mode.

The *ν*_3_ TO mode absorption bands for the 3 : 1, 2 : 1 & 1 : 1 ratios also had a distinct broad, asymmetric profile which reduced in asymmetry for the NH_3_-rich mixtures, but not equivalent to the profile of pure CO_2_ ice. The asymmetry or inhomogeneous broadening of the TO mode absorption band suggested that the absorption band of the C=O asymmetric stretch contained contributions from CO_2_ molecules in different bonding environments. In contrast, the relatively narrow profile of the TO mode absorption band for pure CO_2_ was indicative of a contribution from a largely single bonding environment and previous studies suggested that pure CO_2_ formed dimers upon deposition at 20 K.^18,19^ Where NH_3_ was dominant in the 1 : 10 ratio the CO_2_ molecules were akin to defects in the NH_3_ ice and were essentially matrix-isolated. This reduced CO_2_ dimerisation and thus presented a single bonding environment of the C=O stretch as that of an isolated CO_2_ molecule. While NH_3_ was still in excess in the 1 : 3 ratio, evidence of the formation of CO_2_ : NH_3_ molecular complexes were observed through the absorption band at 3253 cm^−1^. The shoulder on the TO mode absorption band at 2354 cm^−1^ also suggested that there were contributions from two bonding environments to the C=O asymmetric stretch, most likely that of isolated CO_2_ and CO_2_ : NH_3_ molecular complex. This was further supported by the increased asymmetry of the TO mode absorption band for the other mixtures which was likely a combination of several bonding environments that influenced the C=O asymmetric stretch (*e.g.* CO_2_ : NH_3_ molecular complex, CO_2_ dimers and isolated CO_2_ molecules). Therefore the progressive red shift in the peak position with decreasing concentration of CO_2_ reflected the change in asymmetry of the TO mode as a result of different local CO_2_ bonding environment, which arose due to the change in relative CO_2_ and NH_3_ concentrations. The combination modes of CO_2_ were considerably weaker and less sensitive to changes in the lattice compared to the *ν*_3_ asymmetric stretch of CO_2_. The progressive weakening of the CO_2_ combination modes with increasing NH_3_ concentration compared to pure CO_2_ reflected the changes in the CO_2_ bonding environments, as well as the relative decrease in the CO_2_ concentration.

For the NH_3_ vibrational modes in the mixtures (Fig. 2b) a progressive blue shift was observed as the CO_2_ concentration increased (higher *R* values) for the 2*ν*_4_, *ν*_1_ & *ν*_4_ absorption bands. This suggested a progressive strengthening of the N–H bonds with increasing concentrations of CO_2_. At lower concentrations of CO_2_ (1 : 10 & 1 : 3 ratios) the *ν*_3_ absorption band of NH_3_ was red shifted (Δ*ν̃* = −4 cm^−1^). However, a progressive blue shift was observed for increasing concentrations of CO_2_ for the other ratios. In addition, the NH_3_ vibrational modes in the CO_2_ : NH_3_ mixtures also progressively narrowed with increasing concentration of CO_2_ compared to pure NH_3_ absorption bands. Pure NH_3_ ice has extensive intermolecular H-bonding present between the NH_3_ molecules.^20^ For the 1 : 10 ratio where the NH_3_ ice essentially has CO_2_ defects, the intermolecular H-bonding between the NH_3_ molecules was slightly perturbed. As the CO_2_ concentration increased the intermolecular H-bonding was progressively reduced and the formation of CO_2_ : NH_3_ molecular complexes also occurred.

A progressive red shift was observed for increasing concentrations of CO_2_ for the *ν*_2_ absorption band in contrast to the behaviour of the other NH_3_ absorption bands which mainly blue shifted. At higher concentrations of CO_2_, NH_3_ molecules were likely to be bonded in a CO_2_ : NH_3_ complex. The ‘T-shape’ of the CO_2_ : NH_3_ complex resulted in a less restricted bending motion of the *ν*_2_ mode due to reduced intermolecular H-bonding in a CO_2_-rich environment.^14^

### 3.2 Thermal processing

**Fig. 3:**
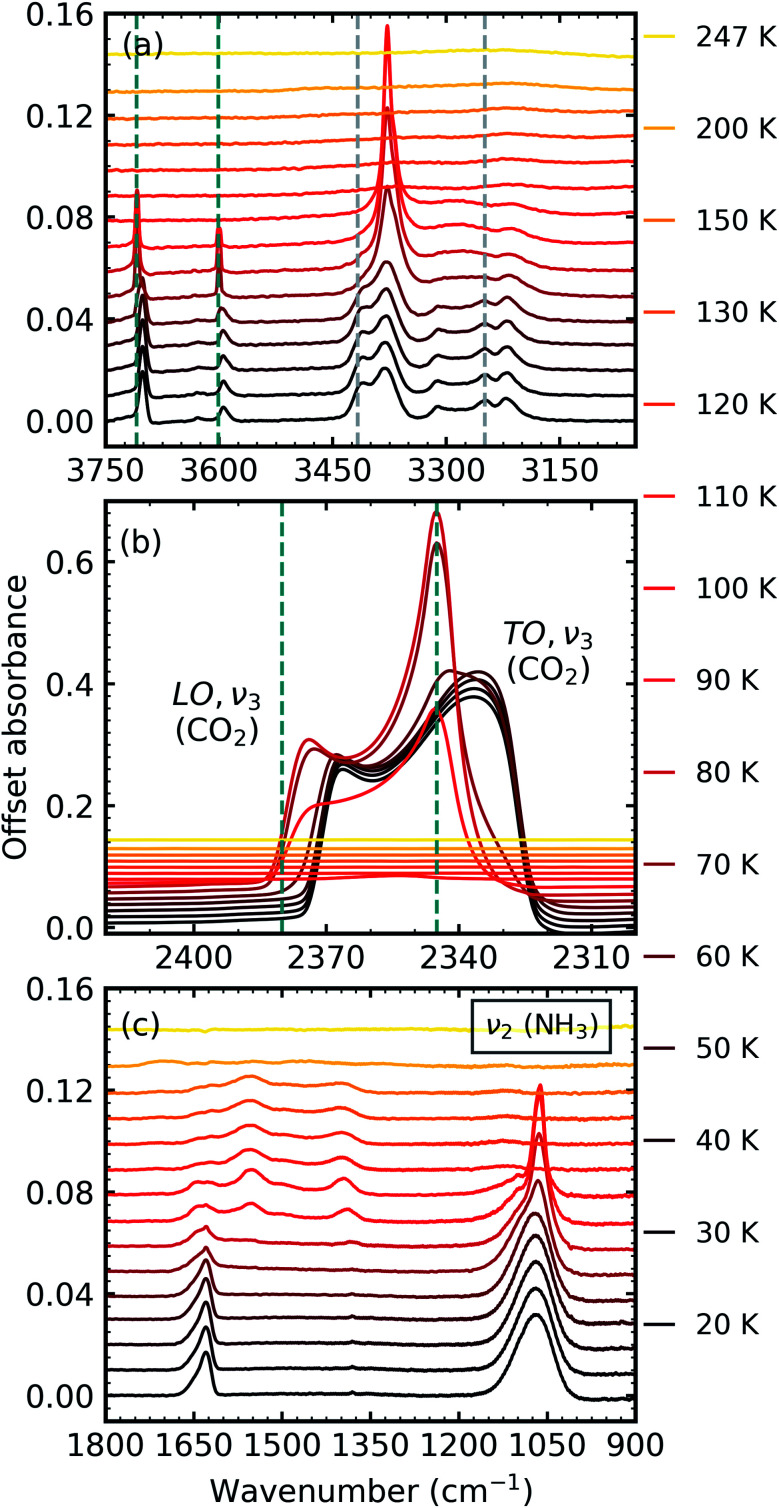
Mid-IR spectra of a CO_2_ : NH_3_ mixture in a 1 : 1 ratio during thermal processing from 20 to 250 K. Spectra are offset on the *y*-axis for clarity. See Section S2 in the ESI† for the mid-IR spectra of the 3 : 1, 2 : 1, 1 : 3 & 1 : 10 ratios. (a) Segregation of the mixture was observed through the shift in the position of the CO_2_ absorption bands towards the position of pure CO_2_ absorption bands when deposited at 20 K which are indicated by blue dashed lines. Grey dashed lines indicate the CO_2_ : NH_3_ molecular complex absorption bands which disappeared between 70–80 K. (b) LO-TO splitting of the *ν*_3_ absorption band of CO_2_. Segregation of the mixture was observed through the shift in the position of the LO and TO modes towards pure CO_2_ positions which are indicated by blue dashed lines. (c) A phase change was observed for NH_3_ through the splitting of the *ν*_2_ absorption band between 70–80 K. New bands between 1800–1200 cm^−1^ at 80 K indicated thermal reaction.

After deposition at 20 K, the CO_2_ : NH_3_ mixtures were thermally processed and analysed at discrete temperatures until desorption. Fig. 3 shows the mid-IR spectra of a CO_2_ : NH_3_ mixture in a 1 : 1 ratio during thermal processing. Mid-IR spectra of the other ratios can be found in Section 2 of the ESI.† For reference, the thermal processing mid-IR spectra of pure CO_2_ and pure NH_3_ are shown in Fig. S8 and S9 of the ESI,† respectively.

Thermal processing induced several changes within the 1 : 1 CO_2_ : NH_3_ mixtures as shown in Fig. 3. Segregation of the homogeneously mixed CO_2_ : NH_3_ ice mixture was identified through a blue shift in the CO_2_ vibrational modes between 60–70 K towards the position of pure CO_2_ vibrational modes when deposited at 20 K (Fig. 3a and b). We note that the only IR absorption band of CO_2_ to shift during thermal processing was the LO mode of the *ν*_3_ asymmetric stretch which reached a maximum blue shift of ∼40 cm^−1^ at 80 K. Splitting of the *ν*_2_ fundamental mode of NH_3_ between 70–80 K signified a phase change in NH_3_ (Fig. 3c). A thermally induced reaction was initiated between 70–80 K and was observed through the appearance of new vibrational modes in the wavenumber region of 1800–1200 cm^−1^ (Fig. 3c). The vibrational modes of the CO_2_ : NH_3_ complex also disappeared between 70–80 K (Fig. 3a). Both CO_2_ and NH_3_ desorbed between 110–120 K leaving behind a residue material which changed between 150–200 K before desorbing by 250 K.

The temperatures at which these changes occurred were dependent on the ratio and are listed in Table 3. Notably, no thermal reaction was observed for the 3 : 1 ratio and the mixture desorbed between 100–110 K.

**Table 3:** Summary of key observations during the thermal processing of CO_2_ : NH_3_ mixtures (3 : 1, 2 : 1, 1 : 1, 1 : 3 & 1 : 10)

Observation	Ratio				
	3 : 1	2 : 1	1 : 1	1 : 3	1 : 10
CO_2_ segregation (K)	By 60	50–60	60–70	70–80	70–80
NH_3_ phase change (K)	By 60	60–70	70–80	70–80	70–80
New bands (K)		>90	>80	>80	>80
New band positions (cm^−1^)				1735	1734
		1647	1647	1646^sh^	1646
		1620	1623	1627	1624^sh^
		1553	1554	1553	1554
			1501^sh^	1501	1506
		1396	1396	1390	1389
Disappearance of CO_2_ : NH_3_ complex (K)	80–90	70–80	70–80	70–80	
CO_2_ desorption (K)	90–100	90–100	100–110	100–110	110–120
NH_3_ desorption (K)	100–110	100–110	100–110	100–110	110–120
Change in residue (K)		150–200	150–200	170–200	170–200
Residue desorption (K)		200–250	200–250	200–250	200–250

#### 3.2.1 Phase change of NH_3_

**Fig. 4:**
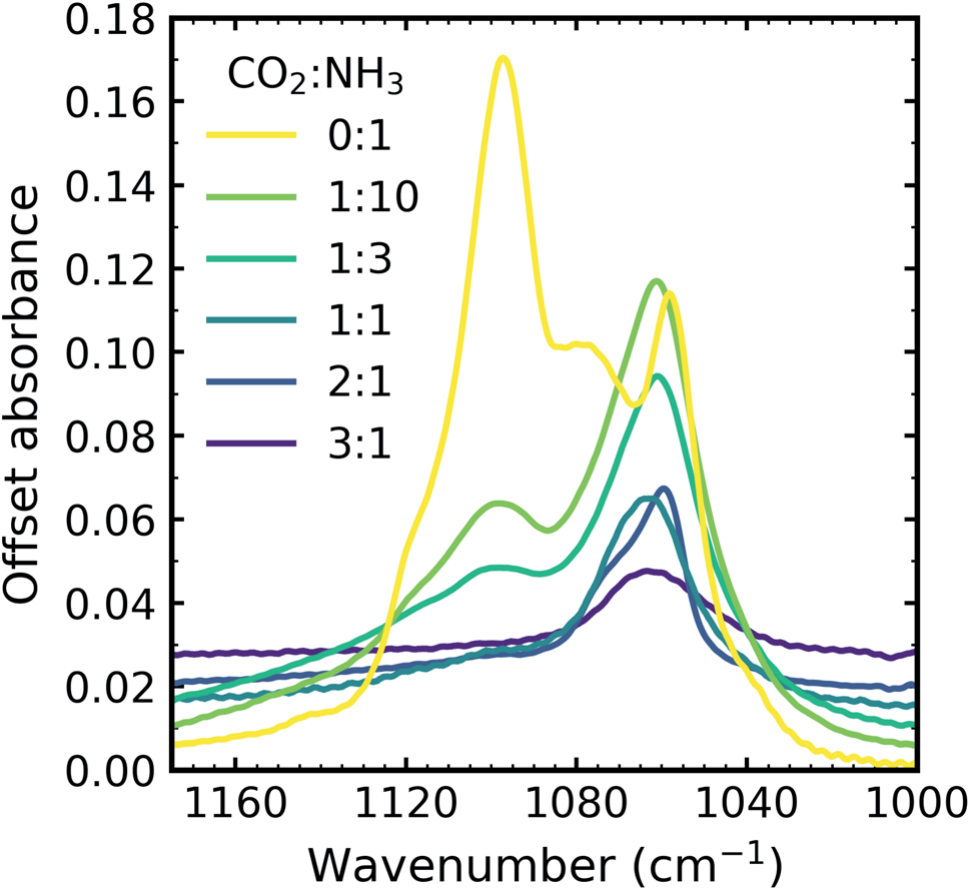
Mid-IR spectra of NH_3_*ν*_2_ umbrella absorption band in the CO_2_ : NH_3_ mixtures which were deposited at 20 K and thermally processed to 90 K compared with pure NH_3_ ice deposited at 20 K and thermally processed to 90 K. Spectra are offset on the *y*-axis for clarity and normalised to a thickness of 400 nm.

Fig. 4 shows the *ν*_2_ absorption band of NH_3_ for all CO_2_ : NH_3_ mixtures and pure NH_3_ deposited at 20 K and thermally processed to 90 K. Different splitting patterns were observed in the *ν*_2_ umbrella absorption band of NH_3_ for the CO_2_ : NH_3_ mixtures compared to pure NH_3_ and also within the mixtures.

Pure NH_3_ deposited at low temperatures (10–20 K) has been described as lacking long-range order or as ‘amorphous’.^20,21^ A more quantitative description has also been presented as containing nano-crystallites comprised of 64 NH_3_ molecules with a lower crystallite size limit of 1.58 nm at 20 K. Depositions at higher temperatures were comprised of larger crystallites (*e.g.* NH_3_ deposited at 60 K formed crystallites comprised of 756(±20%) molecules with a crystallite size of 3.62 nm).^22^ A phase change for pure NH_3_ deposited at 20 K and then thermally processed occurred at 57 K (ref. 21) and was signified by a complex splitting pattern similar to that presented in Fig. 4 for pure NH_3_ (0 : 1). Different splitting patterns have also been observed for NH_3_ deposited between 65–85 K and above 85 K.^20^ The splitting pattern of the 1 : 10 & 1 : 3 ratios were similar to that of pure NH_3_ deposited between 65–85 K and the splitting pattern of the other ratios was similar to that of pure NH_3_ deposited above 85 K.

The bending umbrella motion of the NH_3_ molecules described by the *ν*_2_ vibrational mode was highly perturbed by the surrounding environment. The deposition temperature affected the crystallite size and hence the number of crystallite grain boundaries present. A crystallite grain boundary can be considered as a defect within the ordered H-bonded crystal structure. Broader absorption bands for the *ν*_2_ vibrational mode were observed at lower deposition temperatures of NH_3_ as there were more defects within an ordered H-bonded crystal structure due to the larger surface-area-to-volume ratio of the crystallites. Narrower peaks were observed for NH_3_ deposited at higher temperatures as the crystallite sizes were larger resulting in fewer defects present with the H-bonded crystal (*i.e.* lower crystallite surface-area-to-volume area) and more long-range order. However, thermal processing of the low deposition temperature ices was unable to overcome pre-existing H-bonding within the crystallites that were already formed during deposition, hence forming an ice sample with more crystallite grain boundaries than the equivalent ice deposited at the thermal processing temperature.^20^ This was reflected in the profile of the *ν*_2_ absorption band in Fig. 4 with an extensive splitting pattern resulting from the formation of crystallites of random shapes and sizes.

In Section 3.1 we refer to the presence of CO_2_ within the mixtures disrupting the H-bonding network between NH_3_ molecules. As a consequence, larger NH_3_ crystallites were able to form (with fewer crystallite grain boundaries) in CO_2_ : NH_3_ mixtures with higher concentrations of CO_2_. The difference in the splitting patterns within *ν*_2_ absorption band was a direct effect of the different concentrations of CO_2_ present within the mixtures and we propose that the NH_3_ crystallite formation is dependent on the CO_2_ : NH_3_ ratio.

### 3.3 Residue

A thermal reaction was observed for all CO_2_ : NH_3_ mixtures above ∼80 K, except for the 3 : 1 ratio where no thermal reaction was observed. Previous thermal processing studies of CO_2_ : NH_3_ mixtures have identified ammonium carbamate and carbamic acid as thermal products above 150 K.^3–7^ However, discrepancies exist over the assignment of vibrational modes (see Table S2 in the ESI† for examples). The focus of this study was not to assign all the vibrational modes as this requires complementary theoretical calculations. Instead, differences between the functional groups which compose ammonium carbamate or carbamic acid were used *e.g.* ammonium carbamate is characterised by strong COO^−^ asymmetric and symmetric stretches, while carbamic acid is characterised by C=O and C–O stretches.

**Fig. 5:**
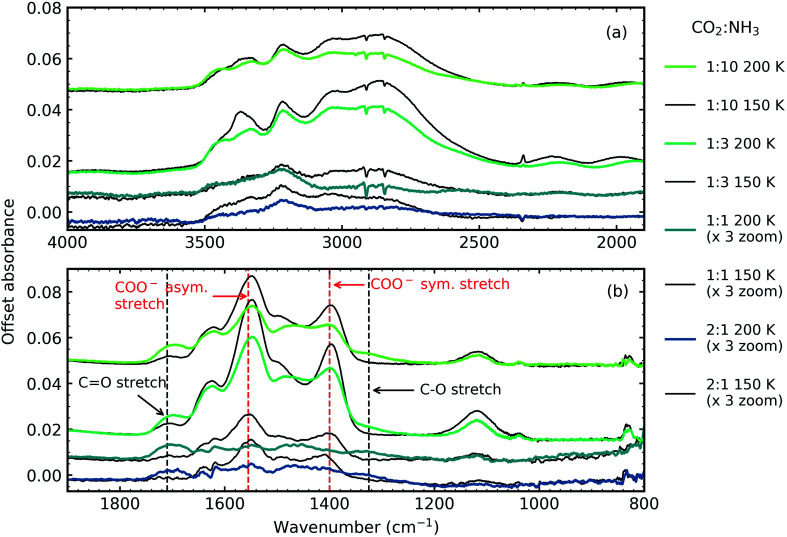
Mid-IR spectra of CO_2_ : NH_3_ ices thermally processed to 150 K (black traces) and 200 K (coloured traces) (a) N–H and O–H stretching region (4000–1900 cm^−1^) and (b) 1900–800 cm^−1^ with dashed lines indicating COO^−^ asymmetric and symmetric stretches and C=O and C–O stretches. The 2 : 1 and 1 : 1 ratios have been magnified by a factor of 3 for clarity. All spectra are normalised to a thickness of 400 nm and offset on the *y*-axis for clarity.

Fig. 5 shows the residue spectra at 150 and 200 K for the 2 : 1, 1 : 1, 1 : 3 and 1 : 10 ratios. The N–H and O–H stretching region is shown in Fig. 5a. Ammonium carbamate and carbamic acid were identified as products in the residue in Fig. 5b at 150 and 200 K and was in agreement with several previous studies.^3,5,6^ The intensity of the residues, and hence the amount of residue formed, was ratio-dependent with the 1 : 2 & 1 : 1 residues significantly weaker in intensity compared to the 1 : 3 & 1 : 10 ratios. The most intense residue was observed for the 1 : 3 ratio similar to a previous study which investigated CO_2_ : NH_3_ mixtures (1 : 1, 1 : 2, 1 : 3) and observed that a 1 : 2 ratio had the largest amount of residue material.^6^ While we also observed a reaction in the 2 : 1 ratio, this residue had the least amount of residue material and no reaction was observed in our 3 : 1 ratio, agreeing with Noble *et al.* that CO_2_-rich mixtures hinder thermal reaction.^7^

Two NH_3_ molecules per one CO_2_ molecule are required to form ammonium carbamate, so it is not surprising that our 1 : 3 ratio had the most intense residue. Yet, the 1 : 10 ratio that was comprised of only 9% CO_2_ produced a more intense residue than the 1 : 1 & 2 : 1 ratios indicating a complex interplay between the different chemical and physical properties of the mixtures and is discussed further in Section 5.

## 4 VUV results & discussion

### 4.1 Deposition at 20 K

**Fig. 6:**
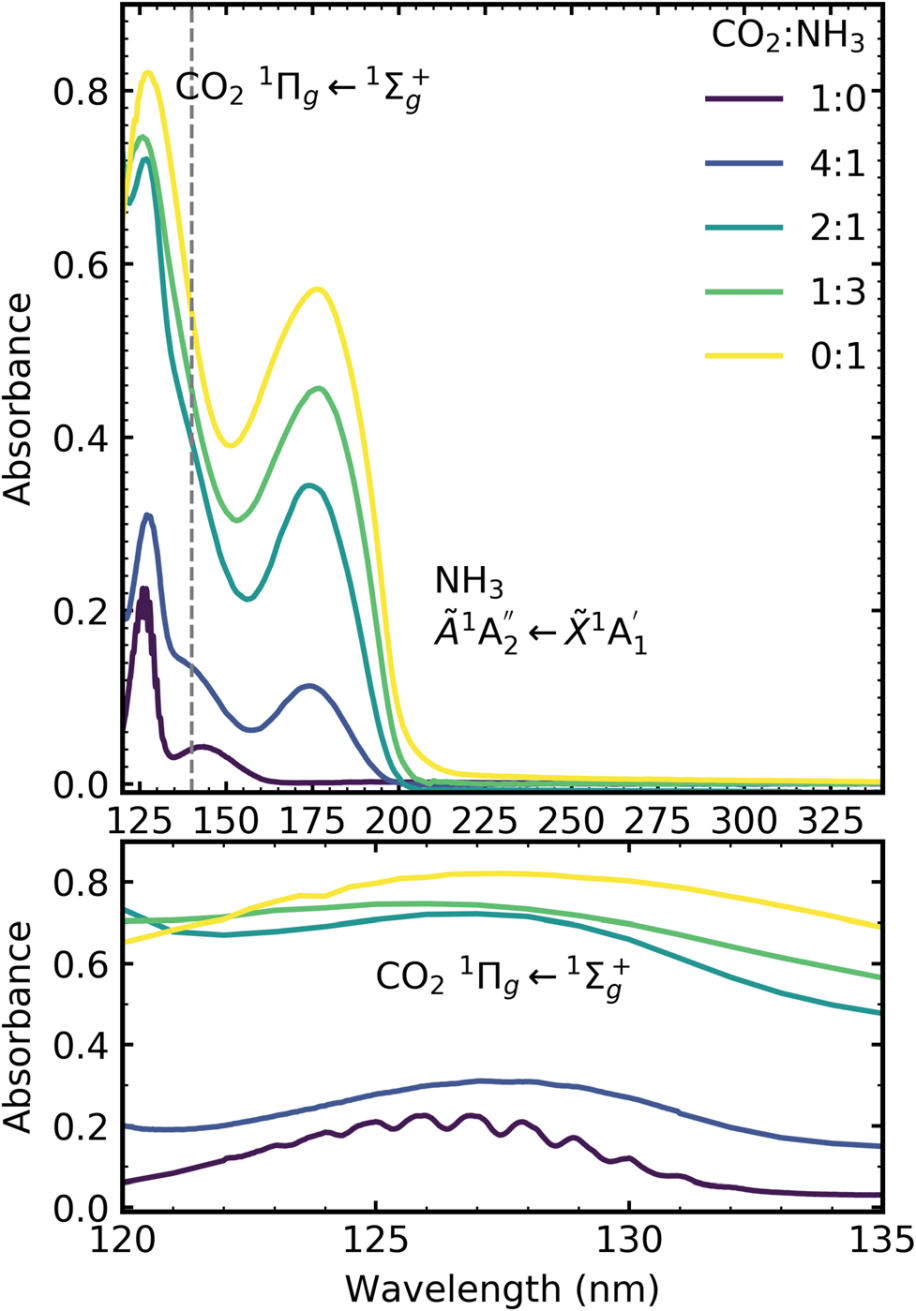
VUV spectra of CO_2_ : NH_3_ mixtures (4 : 1, 2 : 1 & 1 : 3) compared with pure CO_2_ (1 : 0) and pure NH_3_ (0 : 1) between 120–340 nm (top plot). Dashed line highlights the ^1^Δ_u_ ← ^1^Σ^+^_g_ electronic transitions of CO_2_ at ∼140 nm for the 4 : 1 and 2 : 1 ratios. Bottom plot shows the vibrational structure of the CO_2_^1^Π_g_ ← ^1^Σ^+^_g_ transition between 120–135 nm. Spectra are normalised to a thickness of 200 nm.

Fig. 6 shows the VUV spectra of CO_2_ : NH_3_ mixtures (4 : 1, 2 : 1, 1 : 3) deposited at 20 K compared with pure CO_2_ (1 : 0) and pure NH_3_ (0 : 1) also deposited at 20 K.

Pure CO_2_ deposited at 20 K has an absorption band centred around 126 nm due to the ^1^Π_g_ ← ^1^Σ^+^_g_ electronic transition and exhibits extensive vibrational bands.^23^ The average separation between the vibrational bands was 619 cm^−1^ in keeping with previous studies^23–25^ and corresponded to the *ν*_2_ bending mode of CO_2_ suggesting a change in geometry of the molecule from linear to bent upon excitation.^25^ A second, weaker absorption band was observed centred around 143 nm due to ^1^Δ_u_ ← ^1^Σ^+^_g_ electronic transition.^23^ Pure NH_3_ deposited at 20 K has an absorption band centred at 128 nm which was likely due to contributing D, E, F and G ← X Rydberg transitions.^22^ A second, weaker absorption band centred at 178 nm was observed due to the 
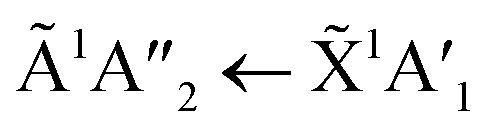
 electronic transition.^20^

The positions of the absorption bands of the ^1^Π_g_ ← ^1^Σ^+^_g_ and ^1^Δ_u_ ← ^1^Σ^+^_g_ electronic transitions of CO_2_ and the 
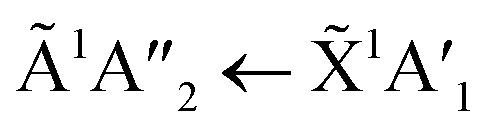
electronic transition of NH_3_ overlap between 120–150 nm. The NH_3_ electronic transitions have a higher cross section compared to that of the CO_2_ electronic transitions and for the 1 : 3 ratio, 
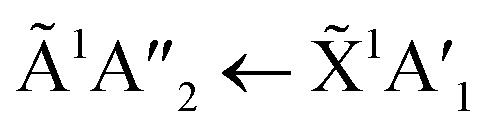
 electronic transition of NH_3_ largely obscures the CO_2_ electronic transitions. For the 4 : 1 & 2 : 1 ratios, the ^1^Π_g_ ← ^1^Σ^+^_g_ electronic transition of CO_2_ was observed centred at 127 nm. Unlike pure CO_2_, no vibrational bands were observed in the ^1^Π_g_ ← ^1^Σ^+^_g_ transition for the 4 : 1 and 2 : 1 mixtures at 20 K. The ^1^Δ_u_ ← ^1^Σ^+^_g_ electronic transition of CO_2_ was observed as a shoulder on the absorption band of the contributing D, E, F and G ← X Rydberg transitions of NH_3_ at 141 nm for the 4 : 1 & 2 : 1 ratios. The 
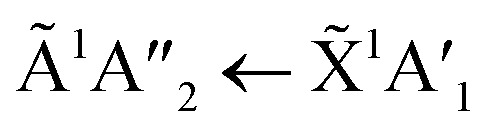
 transition of NH_3_ was observed in all CO_2_ : NH_3_ mixtures and slightly blue shifted compared to the pure NH_3_ band.

### 4.2 Thermal processing

**Fig. 7:**
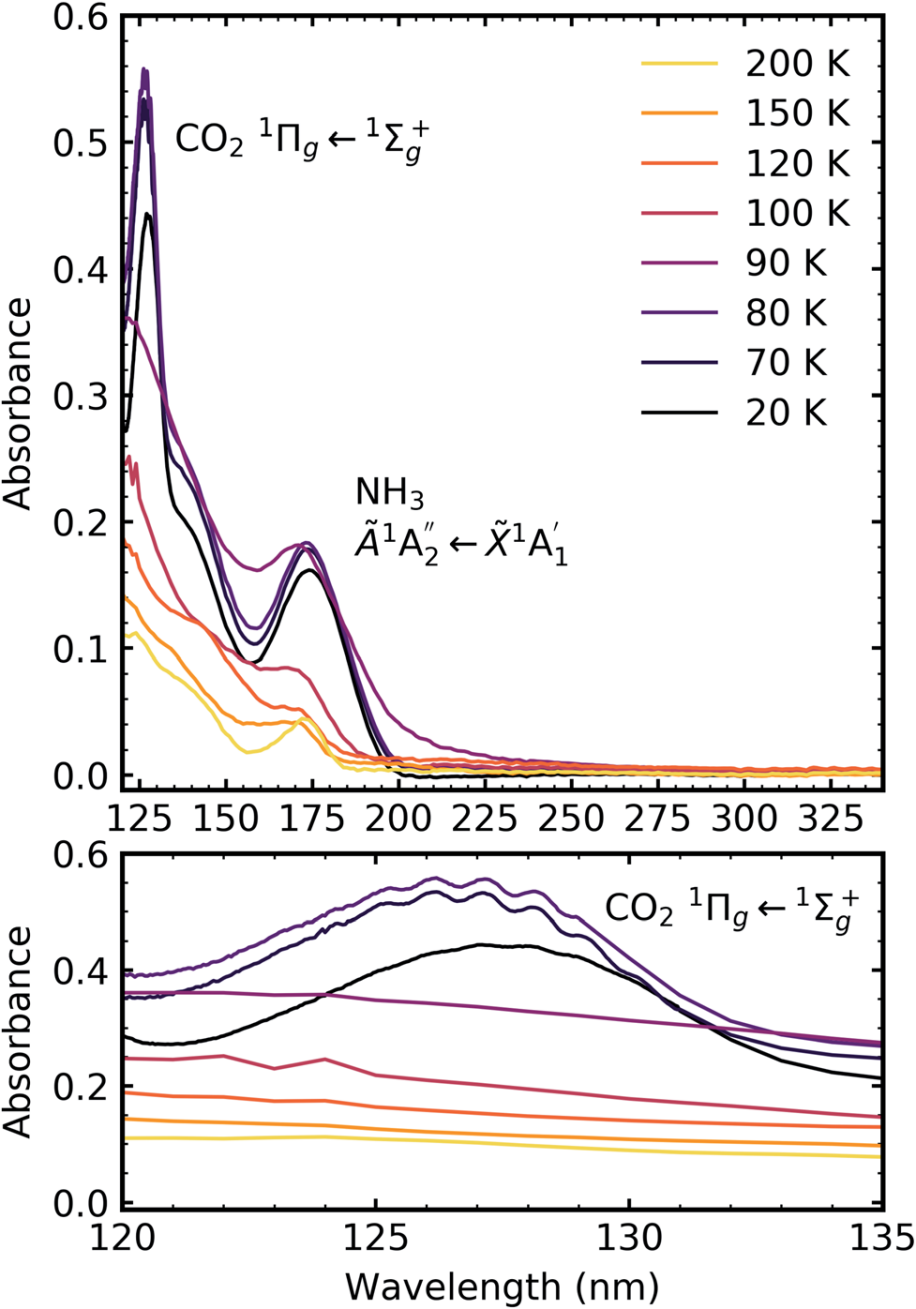
VUV spectra of the thermal processing of a 4 : 1 CO_2_ : NH_3_ mixture from 20–200 K between 120–340 nm (top plot). Bottom plot shows the vibrational structure of the CO_2_^1^Π_g_ ← ^1^Σ^+^_g_ transition between 120–135 nm.

**Fig. 8:**
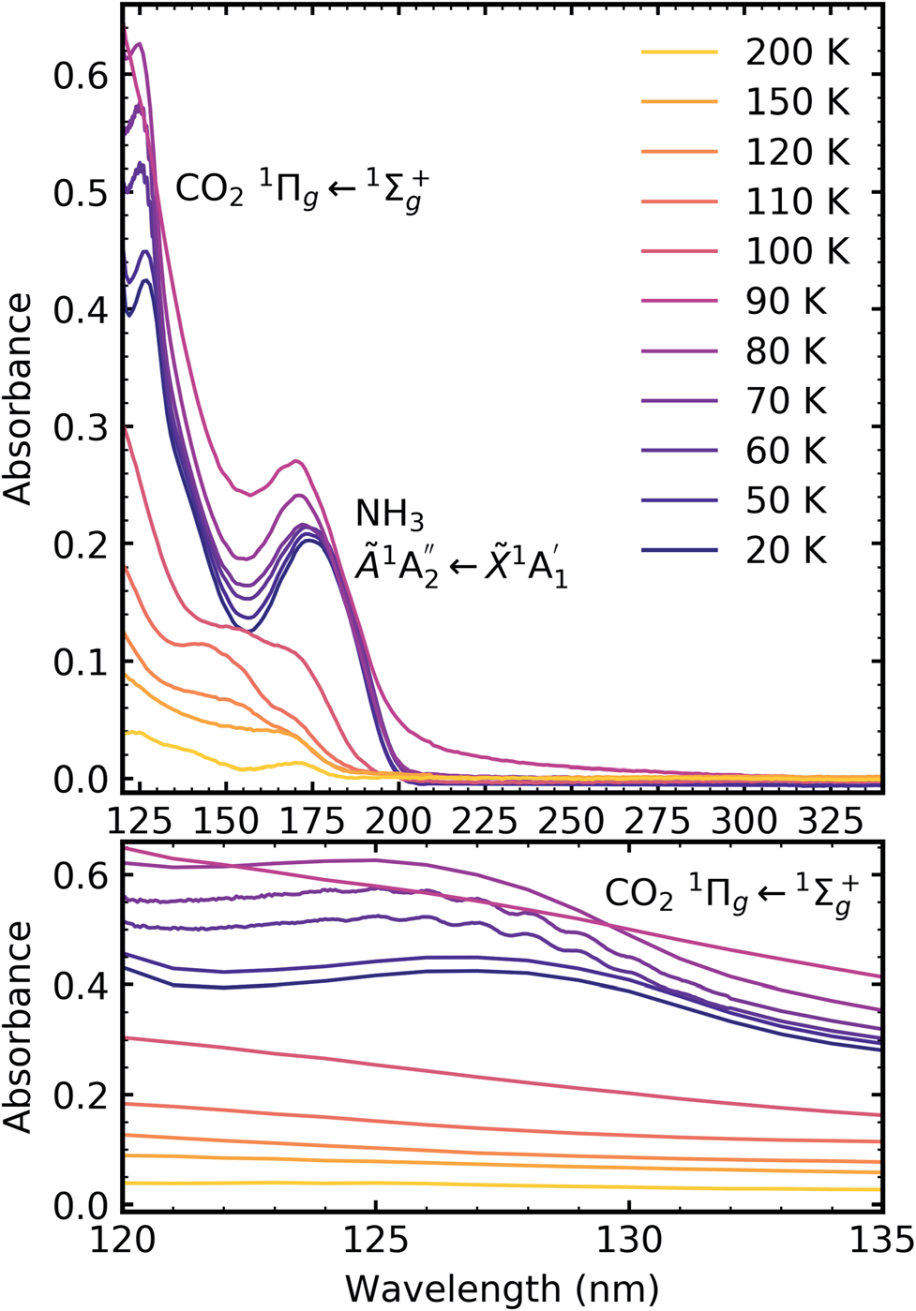
VUV spectra of the thermal processing of a 2 : 1 CO_2_ : NH_3_ mixture from 20–250 K between 120–340 nm (top plot). Bottom plot shows the vibrational structure of the CO_2_^1^Π_g_ ← ^1^Σ^+^_g_ transition between 120–135 nm.

**Fig. 9:**
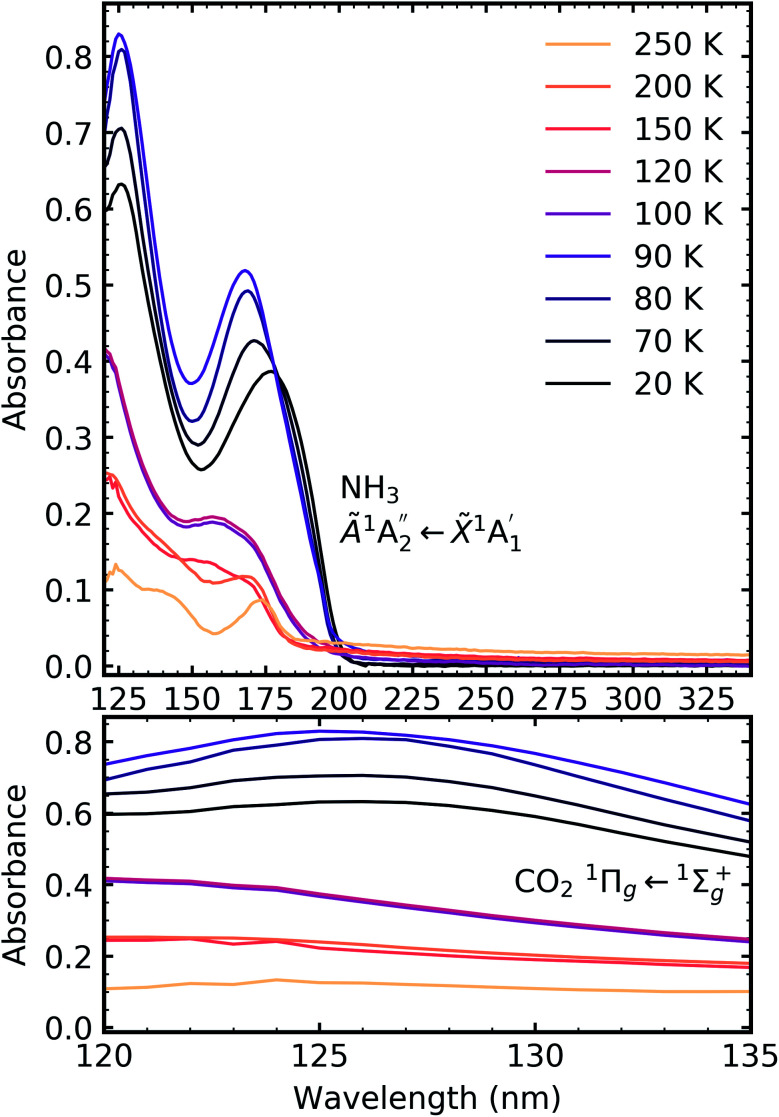
VUV spectra of the thermal processing of a 1 : 3 CO_2_ : NH_3_ mixture from 20–200 K between 120–340 nm (top plot). Bottom plot shows the lack of vibrational structure of the CO_2_^1^Π_g_ ← ^1^Σ^+^_g_ transition between 120–135 nm.

Fig. 7, 8 and 9 show the thermal processing VUV spectra of CO_2_ : NH_3_ mixtures in ratios of 4 : 1, 2 : 1 & 1 : 3 respectively.

Thermal processing VUV spectra of pure CO_2_ and pure NH_3_ ices are shown in Fig. S9 and S10 of the ESI,† respectively and the results are summarised briefly here. For pure CO_2_, vibrational structure was observed on the ^1^Δ_u_ ← ^1^Σ^+^_g_ transition of CO_2_ at 70 and 80 K with an average space separation of 1484 cm^−1^ at 70 K and 1444 cm^−1^ at 80 K. This vibrational structure was not observed at deposition (20 K). For pure NH_3_, factor-group (Davydov) splitting of the 
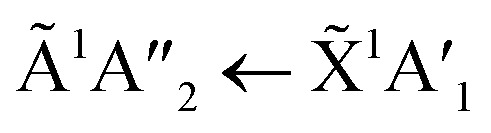
 transition of NH_3_ was observed at 70 K indicating a phase change.^20^

For the CO_2_ : NH_3_ mixtures at 20 K no vibrational structure was observed for the ^1^Π_g_ ← ^1^Σ^+^_g_ transition of CO_2_ for the 4 : 1 & 2 : 1 ratios (obscured in the 1 : 3 ratio). However, vibrational structure was observed upon thermal processing to 70 K for the 4 : 1 ratio and 60 K for the 2 : 1 ratio (VUV spectra below 70 K were not obtained for the 4 : 1 ratio). In agreement with the mid-IR results (see Table 3), desorption of CO_2_ occurred between 90–100 K and was observed through the disappearance of the ^1^Π_g_ ← ^1^Σ^+^_g_ transition for both the 4 : 1 & 2 : 1 ratios.

For all ratios, a blue shift in the 
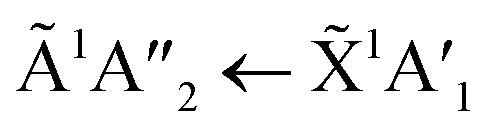
 transition of NH_3_ upon thermal processing was observed. Factor-group splitting observed in the 
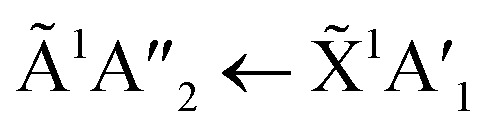
 transition of pure NH_3_ in Fig. S11† of the ESI was not observed in the mixtures. However, a shoulder at 194 nm was observed on the absorption band of the 
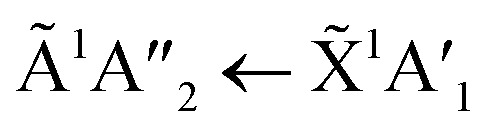
 for the 1 : 3 ratio which was not observed in pure NH_3_ ice, *vide infra*. The 
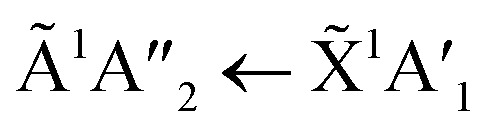
 transition of NH_3_ disappeared by 100 K revealing new absorption bands due to thermal reaction for all ratios including the 4 : 1 ratio where the equivalent mid-IR 3 : 1 ratio did not have an observable residue.

### 4.3 Crystallisation of NH_3_ in the binary mixtures

It was established in Section 3.2.1 that the phase change that occurred between 60–80 K was dependent on the ratio of the CO_2_ : NH_3_ mixtures. Mixtures with higher concentrations of CO_2_ formed larger crystallites and so had a more crystalline structure compared to mixtures with high concentrations of NH_3_.

The subtle morphological changes observed in the mid-IR spectra were more difficult to observe in the VUV spectra. A shoulder observed at 194 nm on the 
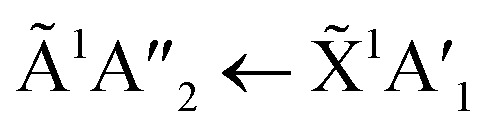
 transition of NH_3_ in the 1 : 3 ratio was assigned as a Wannier–Mott exciton.^20^ Wannier–Mott excitons were observed in NH_3_ ices deposited above 65 K and are linked to the morphology of the ice with it being most prominent in ices with more crystallites.^20^

**Fig. 10:**
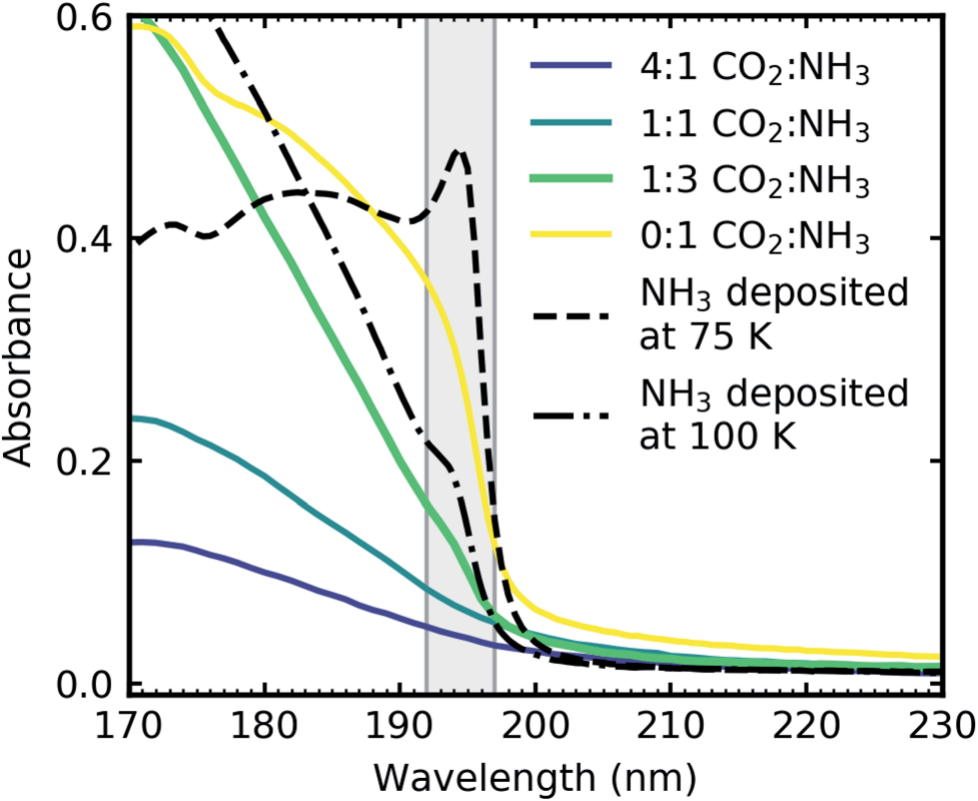
VUV spectra CO_2_ : NH_3_ mixtures (4 : 1, 2 : 1 & 1 : 3) deposited at 20 K and thermally processed to 90 K and pure NH_3_ deposited at 75 K (dash) and 100 K (dash-dot) from Dawes *et al.*^20^ The grey shading indicates the area of the Wannier–Mott exciton which was observed in only the 1 : 3 CO_2_ : NH_3_ mixture (green) and pure NH_3_ deposited at 75 K and 100 K. Spectra are normalised to a thickness of 200 nm and the pure NH_3_ deposited at 75 and 100 K are further scaled by 0.3.

Fig. 10 shows the VUV spectra of the Wannier–Mott exciton peak for the 1 : 3 CO_2_ : NH_3_ mixture compared to Dawes *et al.* data of pure NH_3_ deposited at 75 K and 100 K (ref. 20) and the 2 : 1 & 1 : 3 CO_2_ : NH_3_ mixtures where no Wannier–Mott exciton peak was observed. The exciton peak was much more pronounced for pure NH_3_ ice deposited at 75 and 100 K compared to the exciton peak observed in the 1 : 3 ratio as shown in Fig. 10. The intensity of the exciton peak has been linked to NH_3_–NH_3_ crystallite boundaries which is why we observed an exciton peak in the 1 : 3 ratio. However, the exciton peak in the 1 : 3 ratio was considerably less intense than the exciton peak in pure NH_3_ ices shown in Fig. 10. As the mixture exists as regions of segregated NH_3_ interspersed between regions of segregated CO_2_ ice, there are fewer crystallite grain boundaries for the exciton to propagate similar to a thickness dependence observed in pure NH_3_.^24^

### 4.4 Residue

**Fig. 11:**
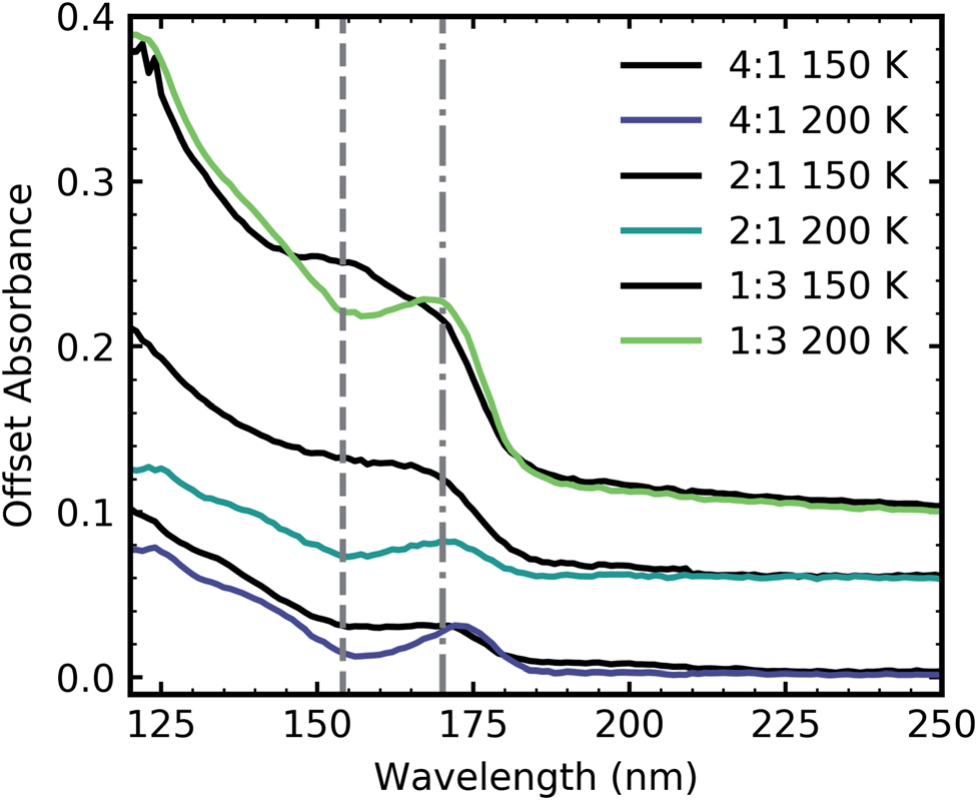
VUV residue spectra of CO_2_ : NH_3_ mixtures (4 : 1, 2 : 1 & 1 : 3) at 150 and 200 K after thermal processing from deposition at 20 K. The dash-dot line highlights a feature at 152 nm and the dashed line highlights a feature at 170 nm. Spectra are normalised to a thickness of 200 nm and offset on the *y*-axis for clarity.

Ammonium carbamate and carbamic acid were identified at 150 K and 200 K for all mid-IR ratios apart from the 3 : 1 ratio (Fig. 5) with evidence of thermal conversion of ammonium carbamate to carbamic acid between 150–200 K. Fig. 11 shows the residue VUV spectra at 150 and 200 K for the 4 : 1, 2 : 1 & 1 : 3 ratios. At 150 K all ratios had a peak at ∼150 nm which decreased upon thermal processing to 200 K. We have tentatively assigned this peak to an electronic transition of ammonium carbamate from the analysis of the mid-IR results which showed that vibrational absorption bands associated with ammonium carbamate decreased between 150–200 K. A dash-dot line in Fig. 11 indicates a shoulder at 170 nm which resolved into a more distinct peak upon thermal processing from 150 to 200 K. We tentatively assigned this to an electronic transition of carbamic acid as mid-IR results indicated that carbamic acid formation increased between 150–200 K.

A noticeable difference between the mid-IR and VUV residue spectra was the presence of a residue for the VUV 4 : 1 ratio. The almost equivalent mid-IR 3 : 1 ratio showed no observable residue. However, a residue material could have been present but in trace amounts below the sensitivity of mid-IR spectroscopy. Even the observed mid-IR 2 : 1 & 1 : 1 residues were significantly less intense than the mid-IR 1 : 3 & 1 : 10 ratios indicating less residue material.

### 4.5 Rayleigh scattering tails

Rayleigh scattering tails have been observed in VUV spectra of several astrophysical ices which did not fully wet the substrate and provided information on the morphology of the ice.^26,27^ Rayleigh scattering tails were observed in the VUV thermal processing spectra (Fig. 7–9). While the ices used in this work were of a thickness where coverage of the surface is expected, if the surface of the ice film was not uniformly smooth then a rough, clumpy surface can scatter the light causing a scattering tail to be observed.

Rayleigh scattering tails occur when particle sizes are less than *λ*/10 and the intensity of scattered light (*I*_s_) is proportional *λ*^−4^ such that:^28,29^

1 *I*_s_ = *I*_0_ × *c* × *λ*^−4^

where *I*_0_ is the incident intensity and *c* is a constant of proportionality which is dependent on the particle size, the refractive index and the number density of scatterers present within the sample.

In the VUV absorption spectra at *λ* > 215 nm, where no absorption peaks were observed, the only contribution to the loss of intensity in transmitted intensity (*I*_t_) was due to scattering. Therefore, by treating the *I*_t_ as equal to *I*_0_ minus *I*_s_ and using the Beer–Lambert Law, the following simple Rayleigh model was fitted:2
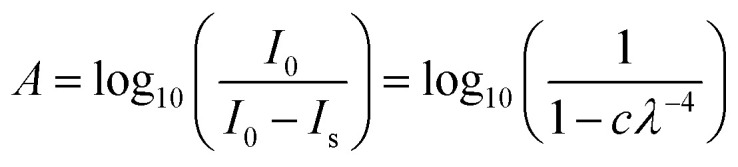


A more meaningful way of representing the changes observed in the scattering from the ice samples is to calculate the fractional change in the constant of proportionality of the processed ice relative to the constant of proportionality at deposition (Δ*c*):3
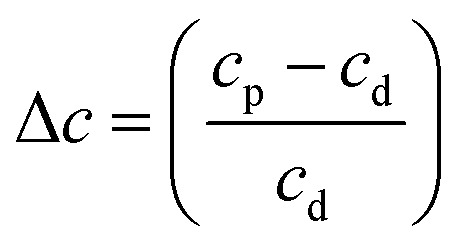
where *c*_p_ is the constant of proportionality of the processed interstellar ice analogue and *c*_d_ is the constant of proportionality of interstellar ice analogue at 20 K.

**Fig. 12:**
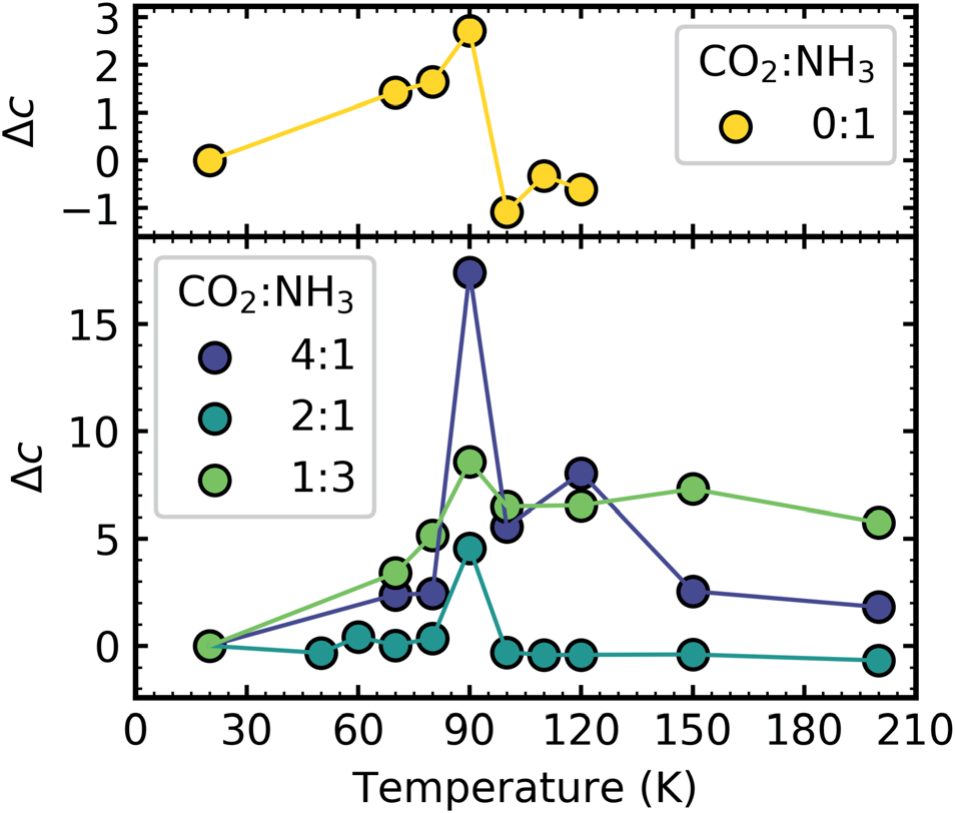
Comparison of the fractional change (Δ*c*) in the constant of proportionality of the processed ice (*c*_p_) relative to the constant of proportionality at deposition (*c*_d_) *versus* thermal processing temperature after deposition at 20 K for pure NH_3_ ice (0 : 1) and CO_2_ : NH_3_ mixtures (3 : 1, 2 : 1, 1 : 1, 1 : 3, 1 : 10). Δ*c* are normalised to a thickness of 200 nm.

Fig. 12 shows the thermal evolution of Δ*c* for pure NH_3_ and the CO_2_ : NH_3_ mixtures. The scattering tails for pure CO_2_ ice were outside the Rayleigh regime. The Rayleigh scattering for pure NH_3_ and the CO_2_ : NH_3_ indicated the presence of particles suggesting that both pure NH_3_ and the mixtures did not cover the substrate as uniformly smooth films and instead formed rough ices with ‘clumps’ on the surface. This probably arose due to the non-wetting behaviour of NH_3_, investigated previously on Au and amorphous water substrates.^30^ Single peaks from temperature-programmed desorption studies indicated that multilayers formed due to the preference of NH_3_ to bind to neighbouring molecules rather than the substrate. While CO_2_ ice is also known to exhibit non-wetting behaviour at low coverages, eventually CO_2_ will cover the entire substrate in a uniform film,^31^ with a smooth surface or with particles that are outside of the Rayleigh regime and no Rayleigh scattering tails could be fitted using eqn (2) for CO_2_ ice.

For pure NH_3_ (0 : 1) and the CO_2_ : NH_3_ mixtures a spike in the Δ*c* value at 90 K was observed. We suggest that the spike in the Δ*c* value for NH_3_ was due to molecular rearrangement and macroscopic change of morphology initiated by the phase change. However, the spike in the Δ*c* value of the CO_2_ : NH_3_ mixtures at 90 K is unlikely to be due to an NH_3_ phase change. Table 3 tells us that the observed phase change of NH_3_ in the CO_2_ : NH_3_ mixtures in the mid-IR study, while ratio-dependent, occurred below 80 K. This was corroborated in Fig. 12 where a slight increase in the value of Δ*c* was observed between 20–80 K. Segregation can also be ruled out as this, while also ratio-dependent, occurred below 80 K. This is further supported by the fact that the largest Δ*c* spike was observed in the 4 : 1 ratio which had segregation and NH_3_ phase temperatures below 60 K. We suggest that the spike in the Δ*c* value was due to CO_2_ desorption. The 4 : 1 and 2 : 1 ratios have the largest amount of CO_2_ in the mixtures and the lowest CO_2_ desorption temperatures (Table 3). Whereas, the CO_2_ in the 1 : 3 ratio desorbed at the same temperature as NH_3_ between 100–110 K likely due to the CO_2_ being embedded within the NH_3_ ice. Desorption of CO_2_ will change the structure of the CO_2_ : NH_3_ mixture and possibly the refractive index of the ice which may cause the spike in Δ*c*. The subsequent macroscopic smoothing of the surface could be attributed to the thermal reaction which is initiated around 80 K causing rearrangement of the ice surface and hence a decrease in the Δ*c* value after CO_2_ desorption.

## 5 Discussion

We set out with the aim of demonstrating the impact that one discrete experimental parameter, the stoichiometric mixing ratio, had on the chemical and physical properties of the CO_2_ : NH_3_ ice system and the subsequent consequence that this may have on thermally induced molecular synthesis.

From our combined mid-IR and VUV spectroscopic results we were able to conclude that CO_2_-rich, equal-parts and NH_3_-rich mixtures had a thermally induced reaction at ∼80 K. A thermally induced reaction at ∼80 K was in agreement with several previous studies which deposited CO_2_ : NH_3_ mixtures at low temperatures (10–20 K).^3,5,6^ For other studies which deposited at higher temperatures^1,2,7^ and/or involved isothermal studies,^8,9^ direct comparison with our results is more difficult when concerning the temperature at which thermal reaction was initiated. For example, Potapov *et al.* reported a lower thermal reaction temperature of 65 K for a KBr substrate.^9^ It is uncertain what the cause of this discrepancy is, although it may be attributed to the different experimental conditions used.

Our residue material at 150–200 K was identified as a mixture of ammonium carbamate and carbamic acid from the literature^3–7^ which underwent a further reaction between 150–200 K as a conversion of ammonium carbamate to carbamic acid.^6,7^ We also identified a ratio-dependence on the amount of residue material present at 150 and 200 K with the largest amount observed in the 1 : 3 ratio and the least amount observed in the CO_2_-richest ratios (*i.e.* mid-IR 3 : 1 and VUV 4 : 1). While it could simply be put that ammonium carbamate was the major product at 150 K and to form it requires two NH_3_ molecules per every one CO_2_ molecule, then stoichiometrically the 1 : 3 ratio was the most ideal. However, we observed differences within both the physical and chemical properties of the CO_2_ : NH_3_ mixtures which provided a comprehensive understanding of how thermally induced molecular synthesis occurred within this system.

We present the first mid-IR study of CO_2_ : NH_3_ mixtures with the substrate at an oblique angle with respect to the IR radiation. This allowed us to probe the LO and TO modes of the *ν*_3_ absorption band of CO_2_ and further characterise the bonding environment of CO_2_ within the mixtures. The absence of a LO mode in the 1 : 10 & 1 : 3 ratio led us to suggest that the CO_2_ molecules were largely matrix isolated in an NH_3_ matrix. Additional vibrational modes associated with the CO_2_ : NH_3_ molecular complex in the 1 : 3 ratio also suggested the presence of CO_2_ : NH_3_ molecular complexes. LO-TO splitting and the asymmetry of the TO absorption bands suggested that the 3 : 1, 2 : 1 & 1 : 1 ratios had bonding environments which included CO_2_ dimers, isolated CO_2_ and CO_2_ : NH_3_ molecular complexes.

**Fig. 13:**
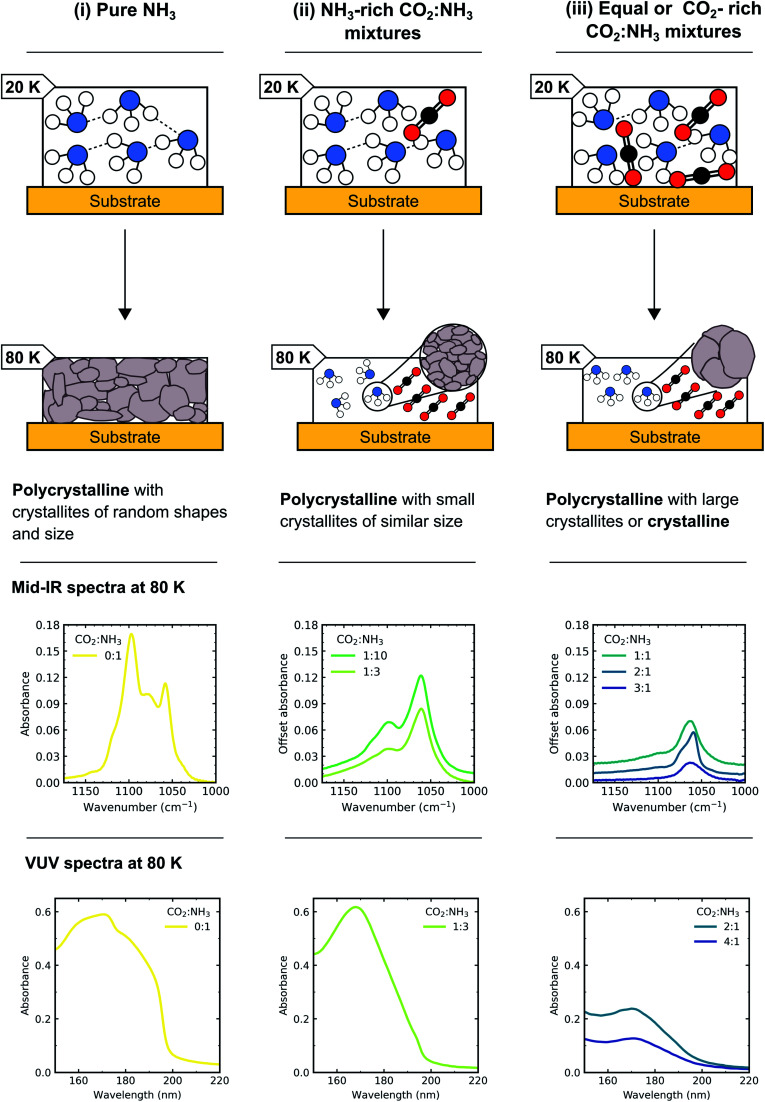
Schematic diagram of how the morphology of NH_3_ changed depending on the ratio of the CO_2_ : NH_3_ mixture. (i) Pure NH_3_ (0 : 1) forms extensive H-bonds at 20 K some of which remain intact upon heating to 80 K so that a polycrystalline structure with crystallites of random shapes and sizes formed. (ii) CO_2_ : NH_3_ 1 : 10 and 1 : 3 ratios formed less extensive H-bonding than pure NH_3_ at 20 K due to the presence of CO_2_ and so upon heating to 80 K, a polycrystalline with small crystallites of similar sizes formed. (iii) CO_2_ : NH_3_ 1 : 1, 2 : 1 and 3 : 1 ratios form even less extensive H-bonding than 1 : 10, 1 : 3 and pure NH_3_ at 20 K due to the presence of CO_2_ and so upon heating to 80 K a polycrystalline with large crystallites or crystalline structure formed. Partly adapted from Dawes *et al.*^20^

This detailed characterisation of the CO_2_ : NH_3_ mixtures at deposition aided our interpretation of the differing NH_3_ crystallite structures observed during thermal processing between the CO_2_ : NH_3_ mixtures. A schematic diagram of the different NH_3_ crystallite structures for pure NH_3_ and the CO_2_ : NH_3_ mixtures is shown in Fig. 13. The NH_3_-rich ratios were identified as having smaller crystallites of similar shapes and sizes (*i.e.* more crystallite grain boundaries) and equal-parts and CO_2_-rich mixtures were identified as having larger crystallites or crystalline structure (*i.e.* less crystallite grain boundaries).

Further physical change in the CO_2_ : NH_3_ mixtures were observed in the Rayleigh scattering tails which tells us that the VUV 4 : 1 ratio underwent a large change in the Δ*c* value at the desorption temperature of CO_2_ at 90 K. This was observed to a lesser extent in the 2 : 1 ratio and only slightly in the 1 : 3 ratio.

If we return to the differing amounts of residue material in the CO_2_ : NH_3_ we now have a deeper understanding of the molecular synthesis occurring within the CO_2_ : NH_3_ mixtures presented in this paper. For example, smaller amounts of residue material were observed in the CO_2_-rich and equal part mixtures. These mixtures mainly consisted of CO_2_ bonded as CO_2_-dimers and to a lesser extent CO_2_ : NH_3_ molecular complexes and isolated CO_2_. Noble *et al.* observed no reaction in their CO_2_-rich mixtures and attributed it to high reaction barriers which caused the CO_2_ to desorb too quickly before a reaction could take place.^7^ While a comparison between our results and Noble *et al.* must be done with caution as they deposited their mixtures at a much higher deposition temperature (60 K) we do also see a large amount of CO_2_ desorption within our CO_2_-rich mixtures. We suggest here that this large desorption of CO_2_ was that of CO_2_-dimers leaving trace amounts of CO_2_ : NH_3_ molecular complexes and isolated CO_2_ to undergo thermal reaction. CO_2_-dimers are less prevalent in the mid-IR 1 : 1 ratio compared to the 2 : 1 (& 3 : 1) ratio which may explain the slightly larger amounts of residue material observed for this ratio. Looking at the NH_3_-rich mixtures, the 1 : 10 ratio consists mainly of isolated CO_2_ and the 1 : 3 ratio consists of isolated CO_2_ and CO_2_ : NH_3_ molecular complexes. This may suggest that the presence of CO_2_ : NH_3_ molecular complexes within the 1 : 3 ratio enhanced the reactivity of the ice.

However, again it was probably not as simple as that. Larger amounts of residue material from the mid-IR study were observed in ratios with more NH_3_ crystallite grain boundaries (1 : 3 & 1 : 10) compared to mixtures with less NH_3_ crystallite grain boundaries (1 : 1 & 2 : 1). In non-astrophysical solids, it is well known that diffusion along crystallite grain boundaries is generally enhanced compared to the crystal or mineral equivalent.^32,33^ Experimental data^34^ and astrochemical models^35,36^ have suggested that structural diffusion within the bulk ice may actually enhance reactivity. This would suggest that CO_2_ diffusion along the crystallite grain boundaries was higher in the NH_3_-rich mixtures which may also have increased reactivity.

## 6 Conclusions

We systematically investigated the stoichiometric mixing ratio in CO_2_ : NH_3_ ices as a function of thermal processing using mid-IR and VUV spectroscopy. This was the first time that CO_2_ : NH_3_ ice mixtures were studied using VUV spectroscopy which revealed a better sensitivity to the residue material. We showed that the CO_2_ bonding environment within the CO_2_ : NH_3_ mixtures were highly dependent on the stoichiometric mixing ratio and that this bonding environment pre-determined the NH_3_ crystallite structures within the CO_2_ : NH_3_ mixtures. By understanding the different chemical and physical properties within the CO_2_ : NH_3_ we were able to extend our understanding of the thermally induced reactions in CO_2_ : NH_3_ mixtures. There was a complex interplay between the different physical and chemical properties of the CO_2_ : NH_3_ mixtures that drove the thermally induced molecular synthesis observed in the CO_2_ : NH_3_ mixtures.

## Conflicts of interest

There are no conflicts to declare.

## Supplementary Material

RA-010-D0RA05826B-s001
