# Supplementary material for: Systematic investigation of CO2 : NH3 ice mixtures using mid-IR and VUV spectroscopy – part 1: thermal processing
Source: RSC Adv. 2020 Oct 12;10(61):37515–28. doi: 10.1039/d0ra05826b (PMC9057146; doi:10.1039/d0ra05826b)
Supplement: RA-010-D0RA05826B-s001 [file RA-010-D0RA05826B-s001.pdf]

Electronic Supplementary Material (ESI) for RSC Advances.
This journal is © The Royal Society of Chemistry 2020

ESI: Systematically investigating CO_2:NH_3 ice mixtures using mid-IR and VUV spectroscopy - Part 1: thermal processing

Rachel L. James, Sergio Ioppolo, Søren V. Hoffmann, Nykola C. Jones, Nigel J. Mason and Anita Dawes

S1 Experimental

S1.1 The Open University Portable Astrochemistry Chamber set-up

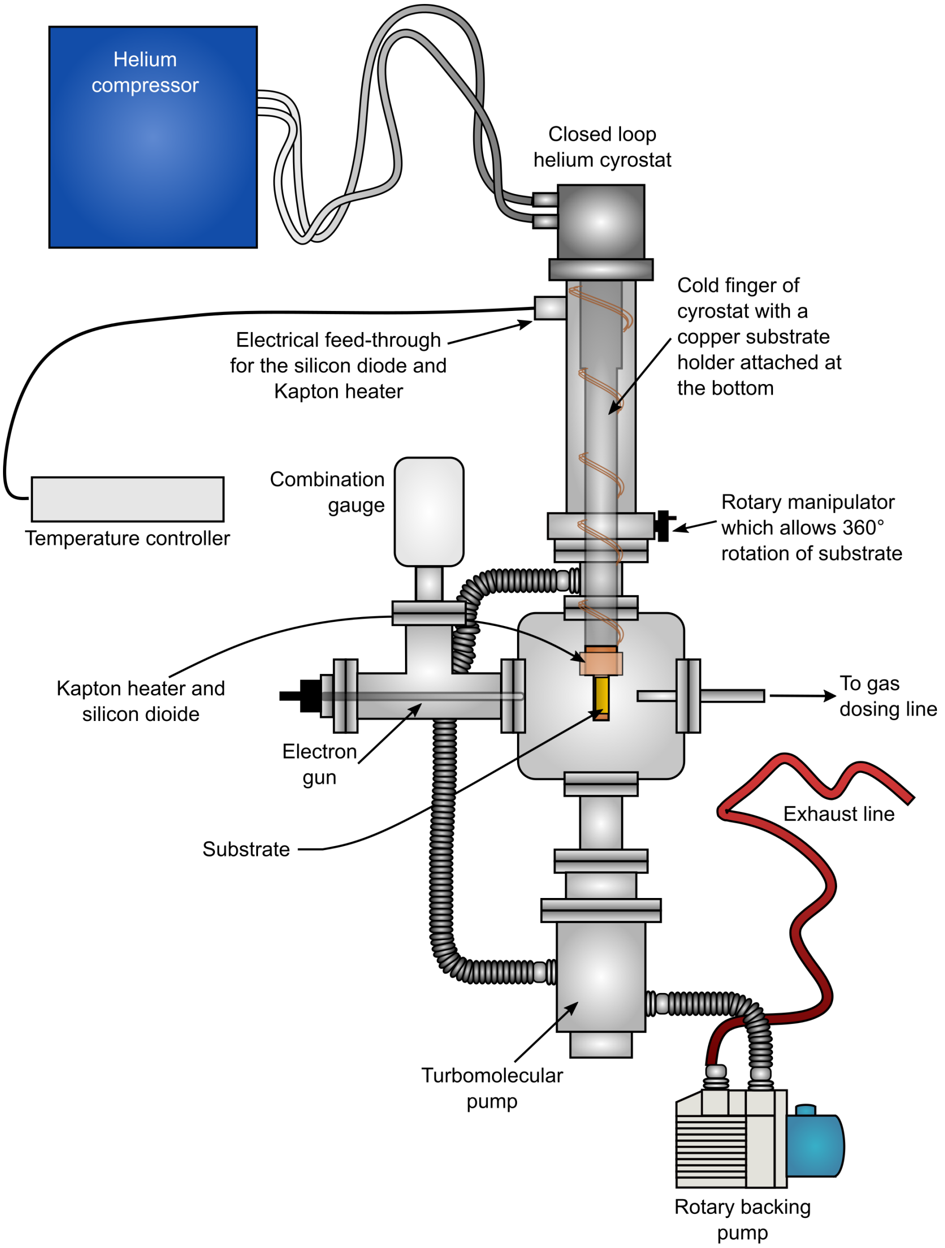

Figure S1 Schematic diagram of The Open University Portable Astrochemistry Chamber (PAC).

Fig. S1 shows a schematic diagram of the Portable Astrochemistry Chamber (PAC). The PAC is a spherical cube chamber (Kimball Physics MCF275-SC600-A) capable of achieving base pressures of low 10^{-9} mbar via a turbomolecular pump (Leybold TURBOVAC TMP 151) backed by a two-stage oil rotary vane pump (Leybold TRIVAC D4B) when at a base temperature of 20 K. The pressure of the chamber was read from a combination gauge (Leybold Ionivac ITR 90) attached to a T-piece onto the chamber which also housed an electron gun which was not used in this study. A substrate holder (OFHC Cu, Goodfellows) attached to the cold head of a two-stage closed cycle helium cryostat (Sumitomo DE-202B) supplied by a helium compressor yielded a base temperature of 20 K. A silicon diode sensor (Lake Shore Cryosensors DT-670B-SD) positioned close to the substrate was used to read the temperature to within ±0.5 K. Thermal processing was achieved via a Kapton flexible resistive heater (Omega) attached to the substrate holder and controlled using a temperature controller (Oxford Instruments Controller ITC 502). A differentially pumped rotary manipulator allowed the substrate holder to be rotated as required.

S1.2 Film thickness

Prior to deposition the substrate was rotated so that it was at normal incidence to the gas inlet nozzle. During deposition the pressure in the PAC was regulated between 1.0–1.2×10^{-7} mbar via a high precision leak valve and *in situ* laser interferometry measurements were taken to estimate the ice thickness.

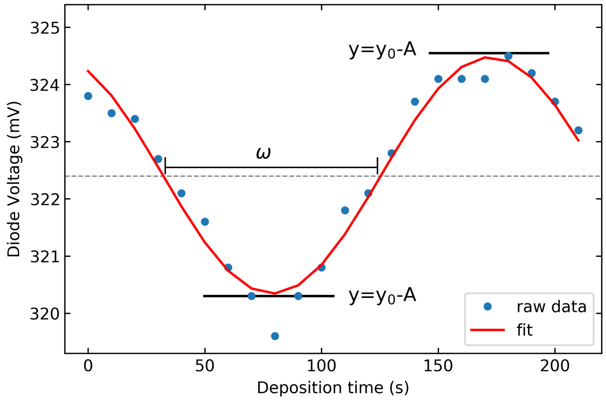

Figure S2 *In situ* laser interferometry voltage measurements against deposition time. The raw data (blue circles) was fitted with Eq. 1 (red line). The y-offset (y_0), phase shift (x_c) half period (ω and amplitude (A) were obtained from the fit.

A HeNe laser beam ($\lambda_0 = 632.8$ nm) was reflected off the surface of the ice sample at an incidence angle of 20° during film growth. Sinusoidal interference patterns due to reflection from the vacuum-ice surface and the ice film-substrate surface were measured with a photodiode and plotted as a function of voltage versus time. An example of this is shown in Fig. S2. The sinusoidal interference pattern was fitted using the following equation:

$$y = y_0 + Asin\left(\pi\frac{x - x_c}{\omega}\right) \tag{1}$$

where y_0 is the y-offset, x_c id the phase shift, *A* is the amplitude and ω is the half period.
The refractive index of the ice film was estimated from the ratio of the minima to maxima of the fitted interference pattern[1]:

$$n_2 = \frac{y_0 + A}{y_0 - A} \tag{2}$$

The angle of the refracted beam was calculated using a rearranged form of Snell's Law:

$$\theta_2 = sin^{-1}\left(\frac{n_1 sin\theta_1}{n_2}\right) \tag{3}$$

where n_1 is the refractive index of the vacuum ($n_1 = 1$), θ_1 is the angle of incidence of the HeNe laser ($\theta_1 = 20°$) and n_2 is the refractive index of the ice film estimated in Eq. 2.
The thickness of the ice per constructive interference fringe (d_f) can then be calculated:

$$d_f = \frac{\lambda_0}{2n_2 cos\theta_2} \tag{4}$$

Table S1 Film thickness of samples used and normalisation factor applied. Mid-IR spectra were normalised to a thickness of 400 nm and VUV spectra were normalised to a thickness of 200 nm.

Spectroscopic study	Ratio	d_s (nm)	Normalisation factor
Mid-IR	1:0	295	1.02
	3:1	380	0.79
	2:1	404	0.74
	1:1	400	0.75
	1:3	424	0.71
	1:10	403	0.74
	0:1	343	1.17
VUV	1:0	308	0.65
	4:1	284	0.70
	2:1	188	1.06
	1:3	140	1.43
	0:1	229	0.87

However, while all ice samples used in this work gave at least one complete interference fringe, as shown in Fig. S2, the sinusoidal interference pattern obtained for the ice sample thickness measurements were not necessarily comprised of an integer value of constructive fringes. Therefore, the deposition rate (r) was calculated using the period (2ω):

$$r = \frac{d_f}{2\omega} \tag{5}$$

Using the deposition time (t), the thickness of the ice sample (d_s) is given as:

$$d_i = \frac{rt}{sin(45)} \tag{6}$$

where sin(45) is the thickness correction due to the substrate being held at a 45° to the IR beam for all mid-IR spectra acquisition.

Table S1 gives the film thickness for pure CO_2, pure NH_3 and CO_2:NH_3 mixtures after deposition at 20 K. A thickness normalisation factor was applied when different ratios were compared.

S1.3 Determining the mid-IR CO_2:NH_3 ratios

The ratios of the CO_2:NH_3 mixtures were determined from the column densities of CO_2 and NH_3 which were calculated from the mid-IR absorption spectra. The column density of molecular species *i* (N_i) is calculated via the following equation:

$$\tau = ln\left(\frac{I_0(\tilde{\nu})}{I_t(\tilde{\nu})}\right) = \sigma(\tilde{\nu})N_i \tag{7}$$

where $\tau(\tilde{\nu})$ is the optical depth at a given wavenumber $\tilde{\nu}$ which is calculated from the incident ($I_0(\tilde{\nu})$) and transmitted ($I_t(\tilde{\nu})$) intensities and $\sigma(\tilde{\nu})$ is the cross section at a given wavenumber $\tilde{\nu}$ (cm^2).

Mid-IR spectra were obtained in absorbance (log scale) and converted to optical depth (ln scale) by multiplication of ln(10). The column density of a specific vibrational mode of molecular species i was then obtained by integrating Eq. 7:

$$N_i = \frac{1}{A_i'}\int \tau(\tilde{\nu})d\tilde{\nu} \tag{8}$$

where $A_i' = \int \sigma(\tilde{\nu})d\tilde{\nu}$ (cm molecule^{-1}) is the infrared apparent band strength of a specific vibrational mode which is constant and obtained from the literature and $\int \tau(\tilde{\nu})d\tilde{\nu}$ is the area of the specific vibrational absorption band.

The CO_2 column density was determined from the ν_3 absorption band near 2340 cm^{-1} using an A_i' of 7.6×10^{-17} cm molecule^{-1} [2] and an integration range between 2400–2300 cm^{-1}. The NH_3 column density was determined from the ν_2 absorption band near 1070 cm^{-1} using an A_i' of 1.63×10^{-17} cm molecule^{-1} [2] and an integration range between 1250–950 cm^{-1}.

We note that the band strength of molecules can be influenced by the matrix in which they reside such that the CO_2 band strength in a 4:1 ratio may be different to the 1:10 ratio. Therefore the A_i' which was obtained from the literature for the pure ices, can introduce errors of up to ±40% uncertainty into the ratio calculation.[3]

S1.4 Determining the VUV CO_2:NH_3 ratios

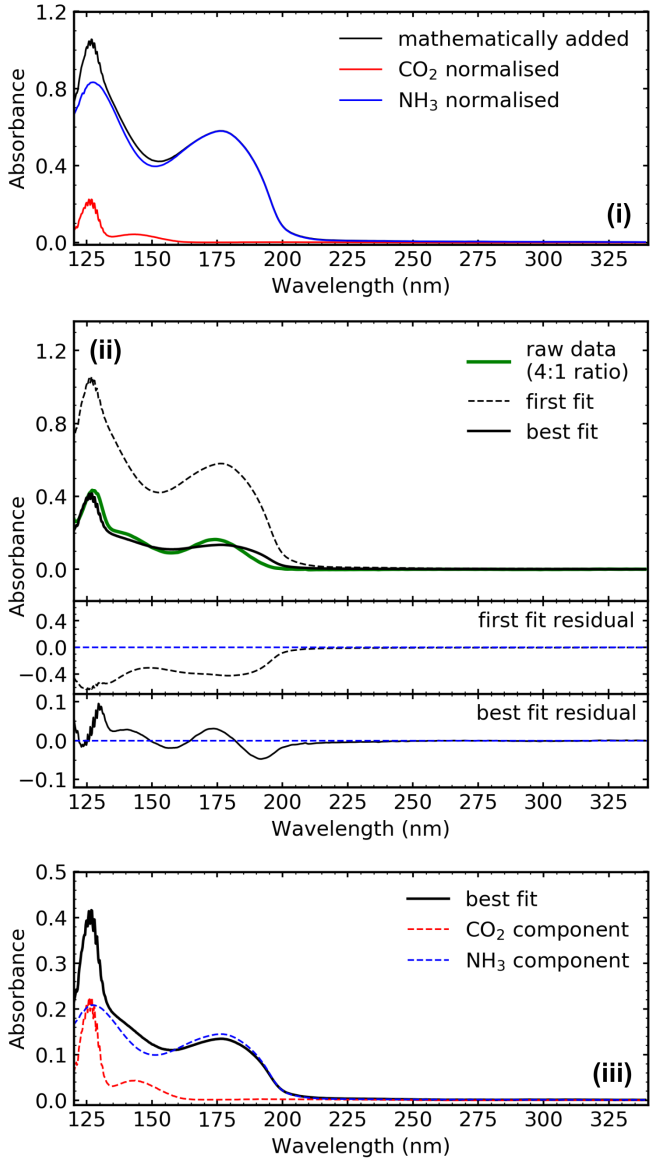

Figure S3 Method for determining the ratio of a CO_2:NH_3 mixture from a VUV spectrum. **(i)** VUV spectra of CO_2 and NH_3 were normalised to the same thickness of 200 nm (red = CO_2, blue = NH_3) and then mathematically added together (black). **(ii)** The first fit (black dashed) was the mathematical spectrum from (i) which was fitted to the raw data (green) and the first fit residual was analysed. The best fit (black solid) was obtained through a manual iterative process which brought the positive and negative components of the residual closest to zero and is shown in the best fit residual. **(iii)** The CO_2 and NH_3 components which make up the best fit can be extracted. In this case the CO_2 component (red dashed) was $\times$ 1.00 that of the normalised CO_2 spectrum and the NH_3 component (blue dashed) was $\times$ 0.23 that of the normalised NH_3 spectrum so an approximate CO_2:NH_3 ratio of 4:1 was obtained.

To determine the CO_2 ratios directly from VUV spectra, the ratios of the CO_2 and NH_3 mixtures were approximated by mathematically adding the VUV spectrum of a pure NH_3 ice and a pure CO_2 ice, analysing the residual and iterating until a best fit was found. A detailed explanation on how the ratios of the VUV spectra were calculated using this approximation is given below:

1. A VUV spectrum of pure CO_2 ice and a VUV spectrum of pure NH_3 ice were normalised to the same thickness, in this case the arbitrary thickness of 200 nm was chosen.
2. The pure spectrum of CO_2 ice and the pure spectrum of NH_3 ice were mathematically added together.

3. The mathematical spectrum was then fitted to a spectrum of a CO_2:NH_3 mixture and the residual was analysed.

4. The best fit was obtained through an iterative process which brought the sum of the positive and negative components of the residual closest to zero. The best fit residual contained both positive and negative components due to shifts in the positions of electronic transition bands and changes in the widths of the electronic transitions bands.

5. From the best fit the CO_2 and NH_3 components were extracted which were given as fractional values of the normalised CO_2 and NH_3 from step 1. The ratio of the CO_2:NH_3 mixture was determined from these fractions.

An example of this method is shown in Fig. S3 for the 4:1 ratio. A ratio of approximately 3:1 was expected for this mixture as during mixing the ratio of the partial pressures of CO_2 and NH_3 mixed in the gas line were the same as the ratio of the partial pressures which were used in the mid-IR study. The other ratios obtained for the VUV spectroscopic study were 2:1 and 1:3.

S2 Additional mid-IR results

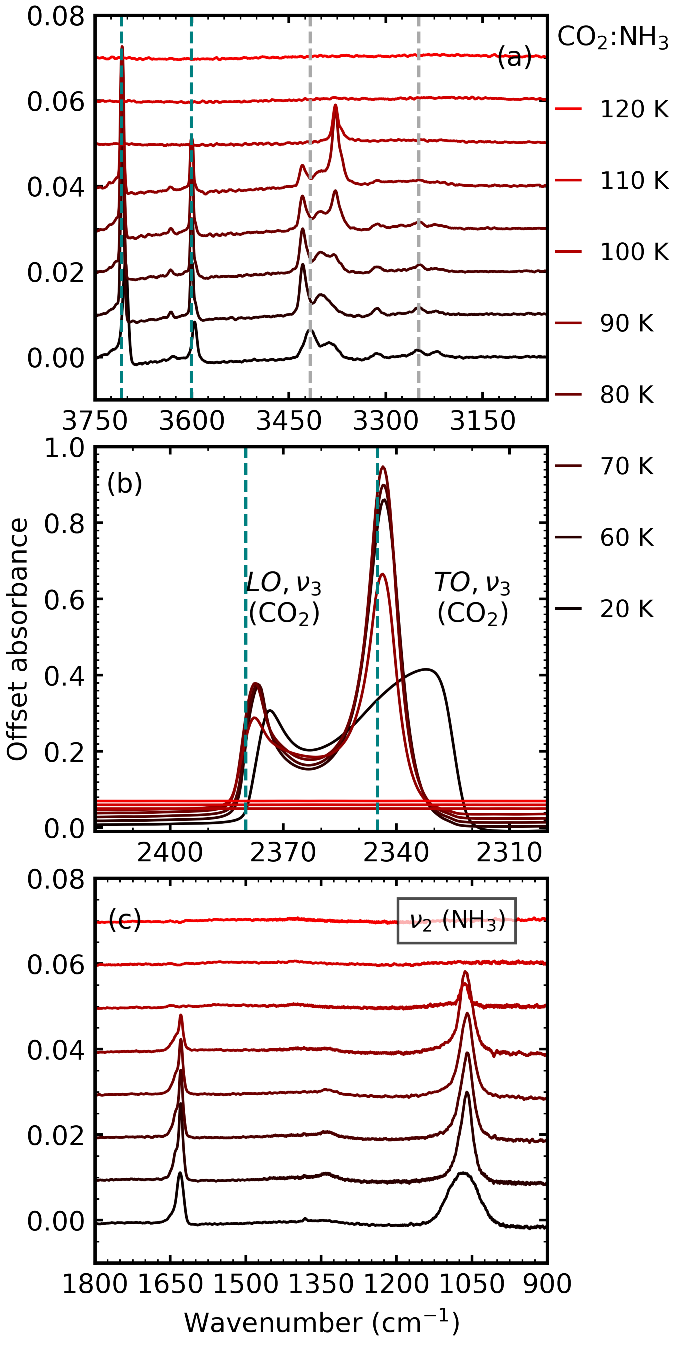

Figure S4 Mid-IR spectra of the thermal processing results of a CO_2:NH_3 mixture in a 3:1 ratio from 20 to 250 K. Spectra are offset on the y-axis for clarity. **(a)** Segregation of the mixture was observed through the shift in the position of the CO_2 absorption bands towards the position of pure CO_2 absorption bands when deposited at 20 K which are indicated by blue dashed lines. Grey dashed lines indicate the CO_2:NH_3 molecular complex absorption bands which disappeared between 70–80 K. **(b)** LO-TO splitting of the ν_3 absorption band of CO_2. Segregation of the mixture was observed through the shift in the position of the LO and TO modes towards pure CO_2 positions which are indicated by blue dashed lines. **(c)** A phase change was observed for NH_3 through the splitting of the ν_2 absorption band between 70–80 K. No new bands observed between 1800–1200 cm^{-1} at 80 K.

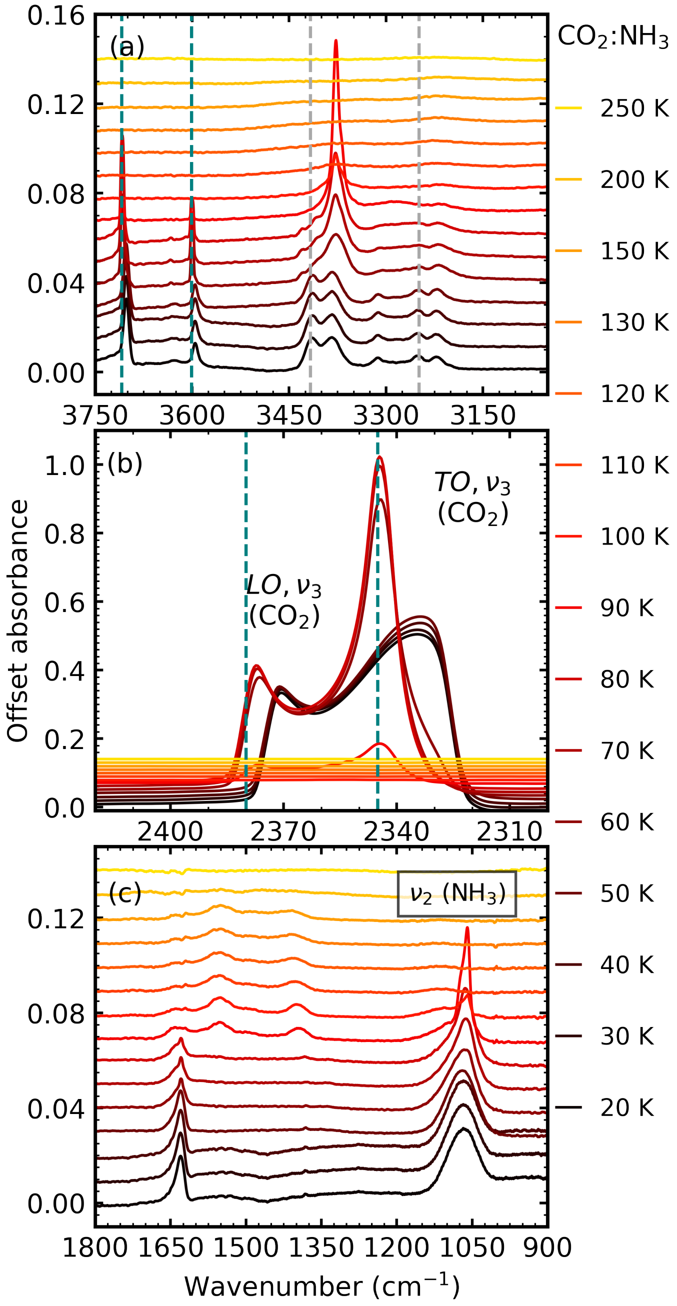

Figure S5 Mid-IR spectra of the thermal processing results of a CO_2:NH_3 mixture in a 2:1 ratio from 20 to 250 K. Spectra are offset on the y-axis for clarity. **(a)** Segregation of the mixture was observed through the shift in the position of the CO_2 absorption bands towards the position of pure CO_2 absorption bands when deposited at 20 K which are indicated by blue dashed lines. Grey dashed lines indicate the CO_2:NH_3 molecular complex absorption bands which disappeared between 70–80 K. **(b)** LO-TO splitting of the ν_3 absorption band of CO_2. Segregation of the mixture was observed through the shift in the position of the LO and TO modes towards pure CO_2 positions which are indicated by blue dashed lines. **(c)** A phase change was observed for NH_3 through the splitting of the ν_2 absorption band between 70–80 K. New bands between 1800–1200 cm^{-1} at 80 K indicated thermal reaction.

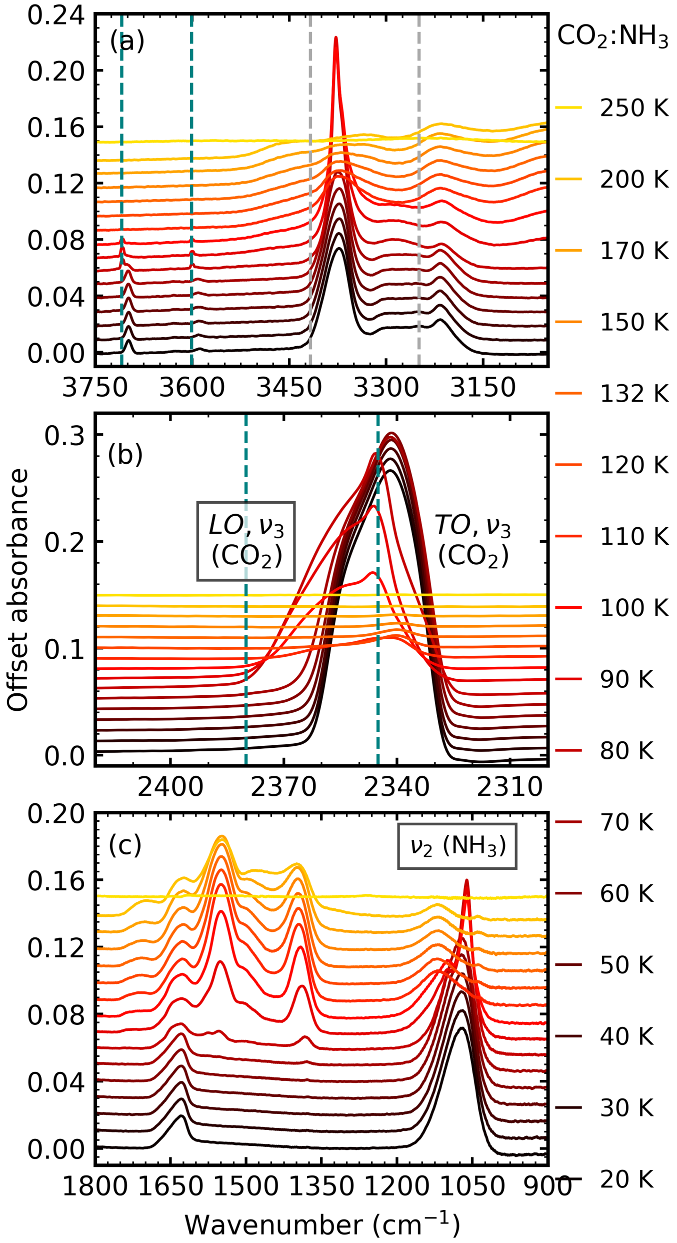

Figure S6 Mid-IR spectra of the thermal processing results of a CO_2:NH_3 mixture in a 1:3 ratio from 20 to 250 K. Spectra are offset on the y-axis for clarity. **(a)** Segregation of the mixture was observed through the shift in the position of the CO_2 absorption bands towards the position of pure CO_2 absorption bands when deposited at 20 K which are indicated by blue dashed lines. Grey dashed lines indicate the CO_2:NH_3 molecular complex absorption bands which disappeared between 70–80 K. **(b)** LO-TO splitting of the ν_3 absorption band of CO_2. Segregation of the mixture was observed through the shift in the position of the LO and TO modes towards pure CO_2 positions which are indicated by blue dashed lines. **(c)** A phase change was observed for NH_3 through the splitting of the ν_2 absorption band between 70–80 K. New bands between 1800–1200 cm^{-1} at 80 K indicated thermal reaction.

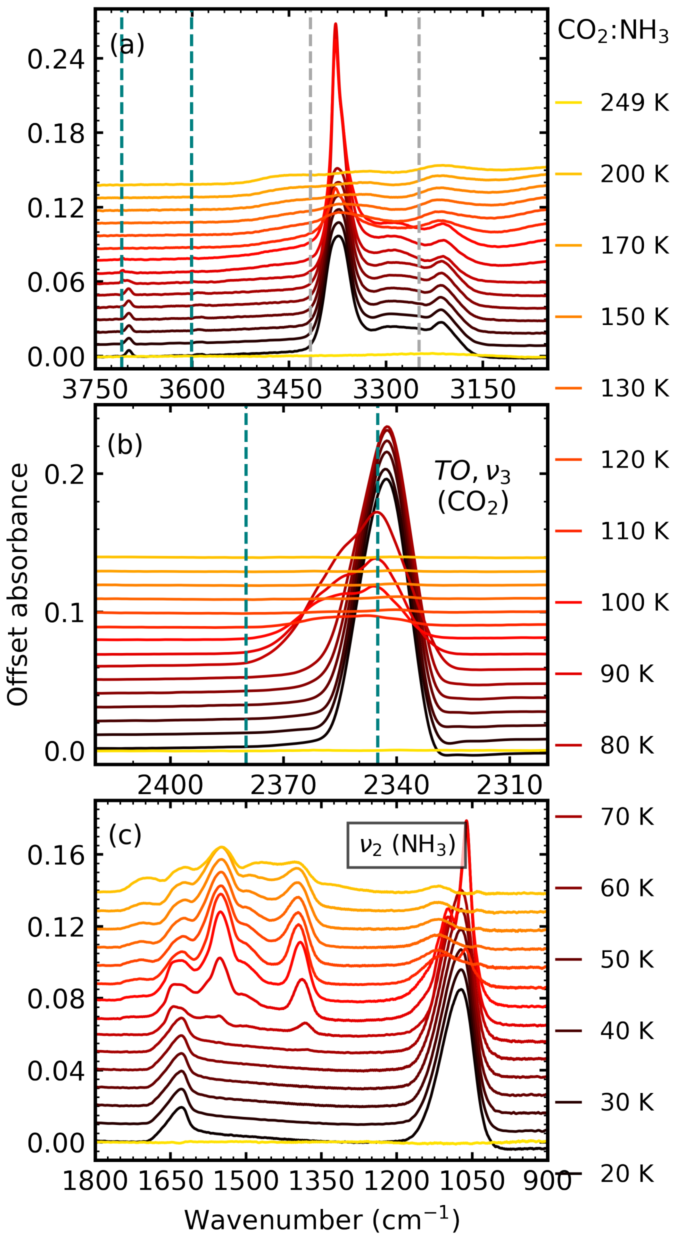

Figure S7 Mid-IR spectra of the thermal processing results of a CO_2:NH_3 mixture in a 1:10 ratio from 20 to 250 K. Spectra are offset on the y-axis for clarity. **(a)** Segregation of the mixture was observed through the shift in the position of the CO_2 absorption bands towards the position of pure CO_2 absorption bands when deposited at 20 K which are indicated by blue dashed lines. Grey dashed lines indicate the CO_2:NH_3 molecular complex absorption bands which disappeared between 70–80 K. **(b)** LO-TO splitting of the ν_3 absorption band of CO_2. Segregation of the mixture was observed through the shift in the position of the LO and TO modes towards pure CO_2 positions which are indicated by blue dashed lines. **(c)** A phase change was observed for NH_3 through the splitting of the ν_2 absorption band between 70–80 K. New bands between 1800–1200 cm^{-1} at 80 K indicated thermal reaction.

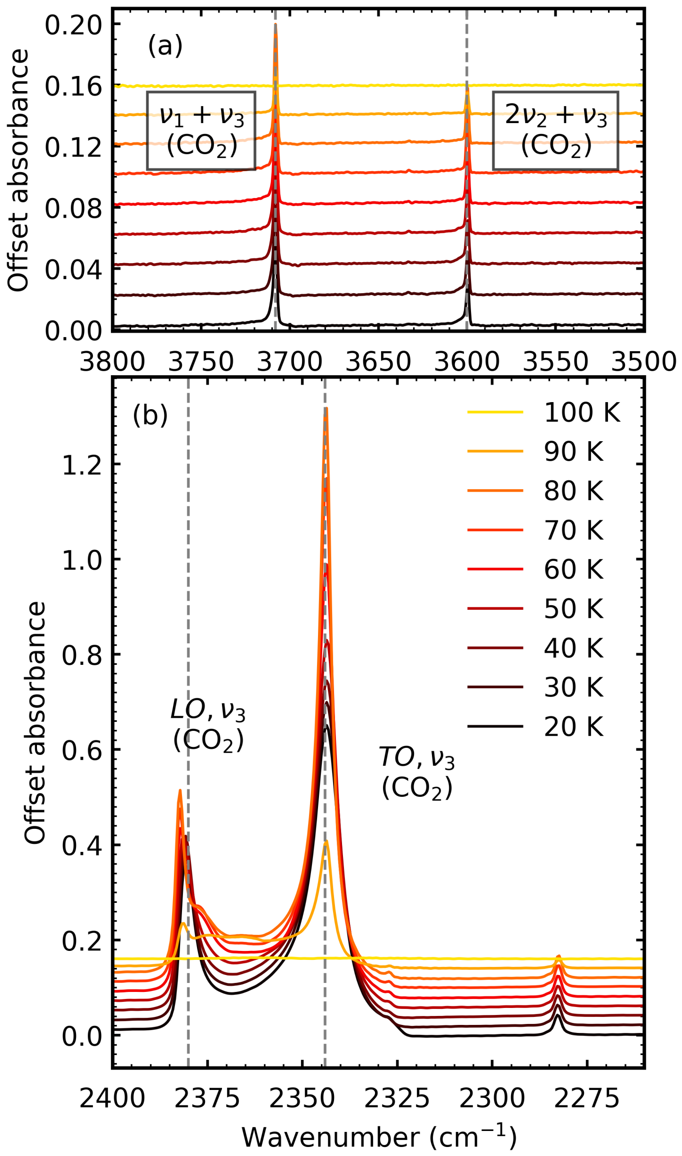

Figure S8 Mid-IR spectra of the thermal processing results of a pure CO_2 ice from 20 to 100 K for **(a)** combination modes and **(b)** LO-TO splitting of the ν_3 absorption band. Dashed lines indicate the position of the CO_2 absorption bands at 20 K. Spectra are offset on the y-axis for clarity.

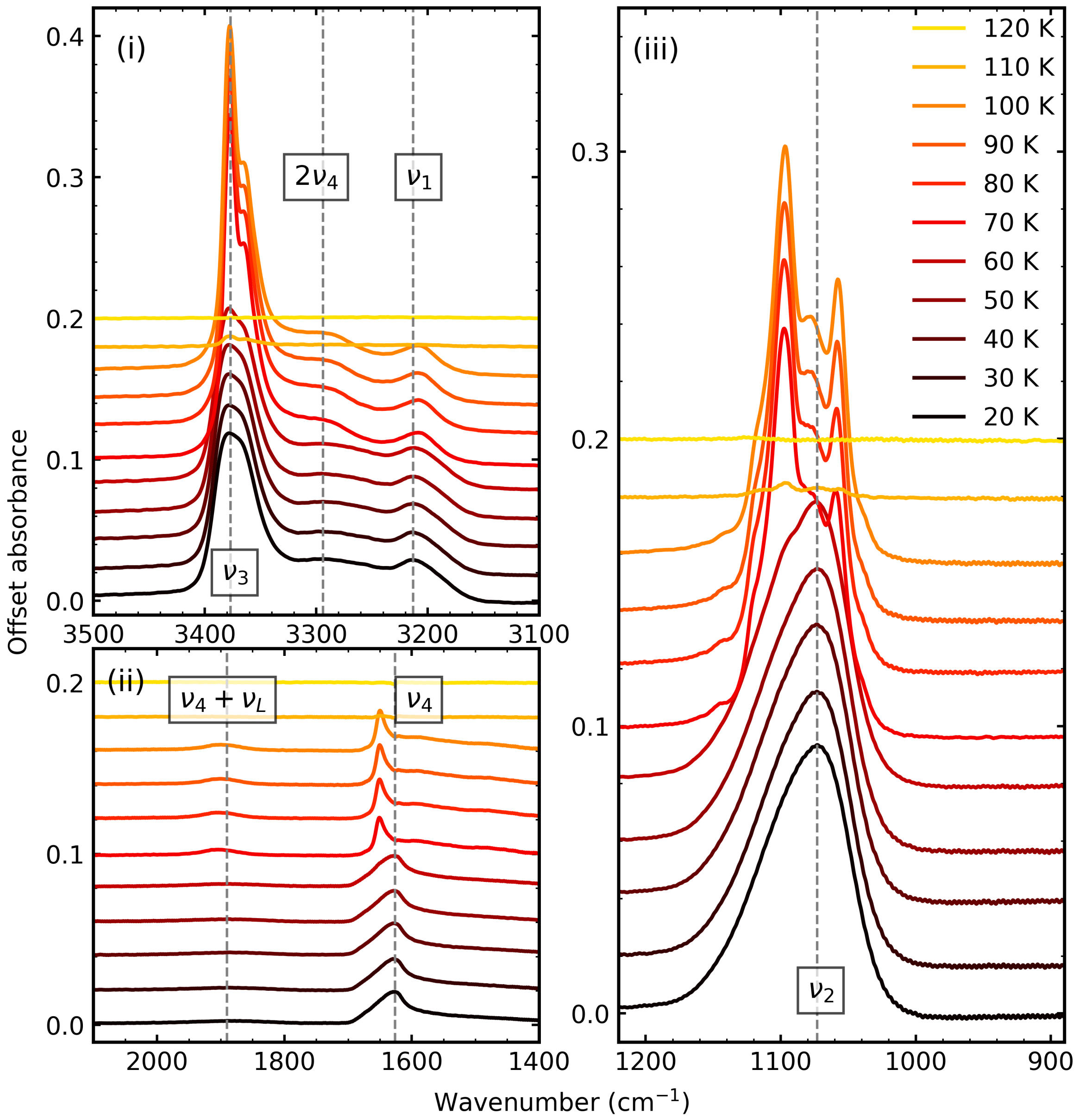

Figure S9 Mid-IR spectra of the thermal processing results of a pure NH_3 ice from 20 to 120 K for **(a)** ν_3, $2\nu_4$ & ν_1 vibrational modes; **(b)** $\nu_4 + \nu_L$ & ν_4 absorption bands and **(c)** ν_2 absorption band. Dashed lines indicate the position of the NH_3 absorption bands at 20 K. Spectra are offset on the y-axis for clarity.

Table S2 Summary of the multiple IR assignments of the same vibrational modes throughout the literature of the thermal processing of CO_2:NH_3 mixtures. * No vibrational mode assigned, asym = asymmetric, sym. = symmetric, oop = out of plane, AC = ammonium carbamate, CA = carbamic acid.

Range (cm^{-1})	Frasco[4]	Hitsatsune[5]	Bossa *et al.*[6]	Rodríguez-Lazcano *et al.*[7]
829–833	NCO_2 oop bend	CO2 wag	OCN oop bend (AC)	NH_2 twist (CA)
1035–1040	NH_2 wag	NH_2 rock	NH_2 wag (AC)	C−N stretch (AC and CA)
1114–1122	CO_2 sym. str.	C−N stretch	C−N stretch (AC)	NH_2 rock (AC)
1261–1265	NH_2 wag	NH_2 rock		
1397–1404	C−N stretch	COO^- sym. Stretch	COO^- sym stretch (AC)	COO^- sym. Stretch (AC)
1445–1451	C−N stretch	${NH_4}^+$ bend	CA dimer	
1460–1500	${NH_4}^+$*	${NH_4}^+$ bend	${NH_4}^+$ bend (AC)	C−O stretch (CA)
1543–1553	COO^- asym stretch	COO^- asym stretch	COO^- asym stretch (AC)	COO^- asym stretch (AC)
1623–1628	N−H bend	N−H bend	N−H bend (AC)	${NH_4}^+$ bend (AC)
1691–1705			C=O stretch (CA)	C=O stretch (AC)
1940–1983	${NH_4}^+$ combination	${NH_4}^+$ stretch		combination (AC)
2172–2236	COO^- sym stretch overtone	${NH_4}^+$ stretch (× 2)		combination (AC)
2747–3089	${NH_4}^+$*	${NH_4}^+$ stretch (× 3)		${NH_4}^+$ stretch (× 2) (AC)
3140 (broad)			O−H stretch (CA)	
3120–3365	N−H sym stretch (× 2)	N−H sym stretch (× 3)	aceNH2 stretch (AC)	2 assignments, NH_2 asym stretch and NH_2 sym stretch (AC)
3415–3475	N−H asym stretch	N−H asym stretch	NH_2 stretch (AC and CA)	NH_2 asym stretch or O-H stretch (AC or CA)

S3 Additional thermal processing VUV spectra

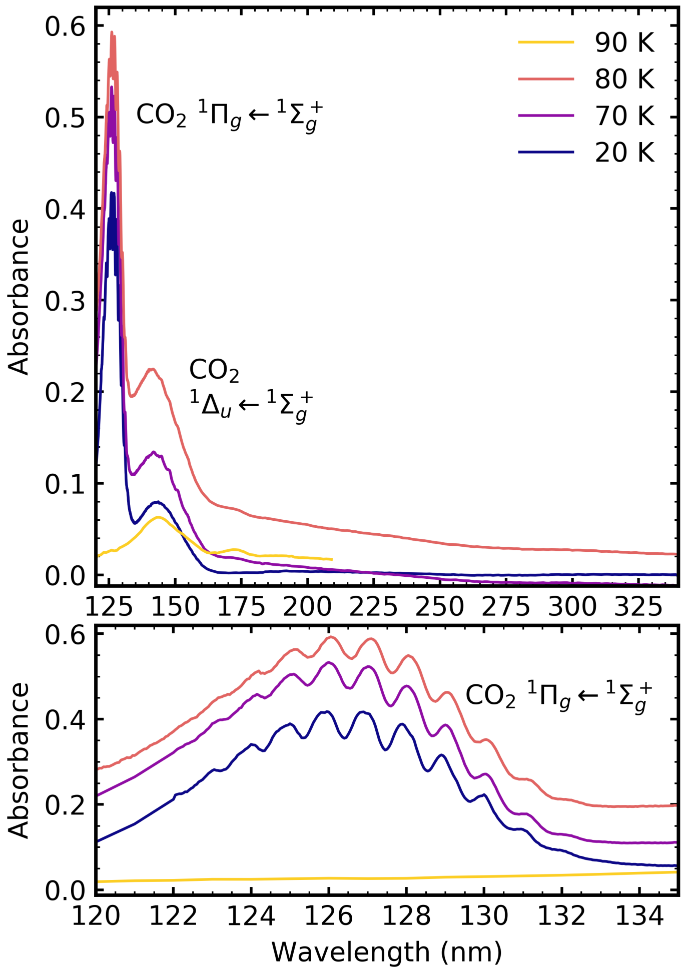

Figure S10 VUV spectra of the thermal processing of a pure CO_2 mixture from 20–90 K between 120–340 nm (top plot). Bottom plot shows the vibrational structure of the CO_2 $^1\Pi_g \leftarrow^1 \Sigma_g^+$ transition between 120–135 nm. Note, at 90 K CO_2 was desorbing and absorption spectra was only obtained between 120–220 nm.

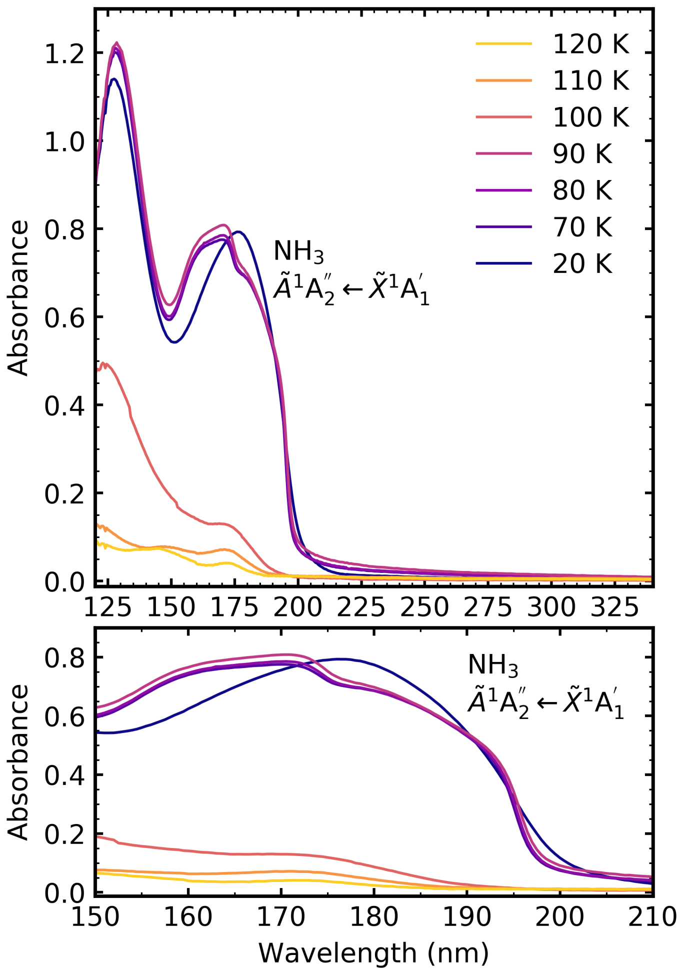

Figure S11 VUV spectra of the thermal processing of a pure NH_3 mixture from 20–120 K between 120–340 nm (top plot). Bottom plot NH_3 $\tilde{A}^1A_2'' \leftarrow \tilde{X}^1A_1'$ transition between 150–210 nm.

Notes and references

[1] M. S. Westley, G. A. Baratta and R. A. Baragiola, *J. Chem. Phys.*, 1998, **108**, 3321–3326.

[2] M. Bouilloud, N. Fray, Y. Bénilan, H. Cottin, M.-C. Gazeau and A. Jolly, *Mon. Not. R. Astron. Soc.*, 2015, **451**, 2145–2160.

[3] J. Terwisscha van Scheltinga, N. F. W. Ligterink, A. Boogert, E. van Dishoeck and H. Linnartz, *Astron. Astrophys.*, 2018, **611**, A35.

[4] D. Frasco, *J. Chem. Phys*, 1964, **41**, 2134–2140.

[5] I. C. Hisatsune, *Can. J. Chem*, 1983, **62**, 945–948.

[6] J. B. Bossa, F. Duvernay, P. Theulé, F. Borget and T. Chiavassa, *Chem. Phys.*, 2008, **354**, 211–217.

[7] Y. Rodríguez-Lazcano, B. Maté, V. J. Herrero, R. Escribano and Ó. Gálvez, *Phys. Chem. Chem. Phys*, 2014, **16**, 3371–3380.
